# Effectiveness of Gamified Swallowing Exercises in Adults With Dysphagia: Systematic Review and Meta-Analysis of Randomized Controlled Trials

**DOI:** 10.2196/82017

**Published:** 2026-03-26

**Authors:** Jijia Liu, Meijun Ou, Mengyao Yan, Xianghua Xu, Li Qin

**Affiliations:** 1 The Affiliated Cancer Hospital of Xiangya School of Medicine, Central South University/Hunan Cancer Hospital, 283 Tongzipo Road, Yuelu District, Changsha, 410013, China, 86 13973177554; 2 Xiangya School of Nursing, Central South University, Changsha, China; 3 The 921st Hospital of Chinese People's Liberation Army (Second Affiliated Hospital of Hunan Normal University), Changsha, China

**Keywords:** deglutition disorders, gamified, swallowing exercise, systematic review, meta-analysis

## Abstract

**Background:**

Dysphagia is a prevalent health issue affecting quality of life. Gamified swallowing exercises have the potential to enhance swallowing function and adherence in adults with dysphagia. Nevertheless, such evidence has not yet been systematically synthesized.

**Objective:**

This study aimed to systematically evaluate the effects of gamified swallowing exercises and promote their clinical application in swallowing rehabilitation.

**Methods:**

Eleven electronic databases were searched from inception to June 25, 2025. Randomized controlled trials (RCTs) that explored gamified swallowing exercises in adults with dysphagia, regardless of etiology, and reported on swallowing function, adherence, nutritional status, or quality of life were included. The Cochrane Risk of Bias tool 2.0 was applied to assess the methodological quality. We employed the Hartung-Knapp-Sidik-Jonkman method for random-effects model analysis to calculate effect sizes and 95% CIs. Subgroup analysis was conducted to explore potential causes of heterogeneity, and the overall quality of evidence was evaluated through GRADEpro GDT software.

**Results:**

Of 2400 initially identified records, 6 RCTs met the inclusion criteria, with 2 rated as low risk, 3 as some concerns, and 1 as high risk. These studies, conducted in China, South Korea, and Turkey between 2019 and 2025, enrolled a total of 330 poststroke patients with dysphagia. Interventions involved 1 to 3 games targeting the lips, tongue, and pharynx, with additional equipment. This meta-analysis indicated that gamified swallowing exercises improved swallowing function, as evidenced by enhanced swallowing performance (mean difference [MD]=1.1; 95% CI 0.9 to 1.3; *I*^2^=0; prediction interval [PI] 0.3 to 1.6), reduced dysphagia severity (standardized mean difference [SMD]=0.4; 95% CI 0.3 to 0.5; *I*^2^=0; PI 0.3 to 0.5), and increased adherence (MD=2.4; 95 % CI 1.8 to 2.9; *I*^2^=0; PI 1.2 to 2.7). However, no significant effects were observed on dysphagia screening (SMD=−1.8, 95% CI −5.4 to 1.8; *I*^2^=96.5%; PI −8.6 to 4.9) or quality of life (SMD=−2.30, 95% CI −9.6 to 5; *I*^2^=97.5%; PI −16.1 to 14.1). Overall, the quality of the included studies ranged from moderate to very low, which limits confidence in the pooled estimates.

**Conclusions:**

This study provides the first quantitative synthesis of the effects of gamified swallowing exercises. The results demonstrate that gamified swallowing exercises improve swallowing function and enhance adherence, suggesting that gamified swallowing exercises are a promising method for swallowing rehabilitation. However, the overall risk of bias across included studies and the suboptimal evidence quality reduce the certainty of the current evidence. Therefore, the findings should be interpreted with caution. The small number of high-quality RCTs, reliance on additional equipment, and limited standardization of gamified interventions further limit clinical generalizability. Future research should focus on large-scale, robustly designed RCTs, while designing etiology-specific games and developing more accessible rehabilitation equipment, such as smartphones, could enhance the overall effects and facilitate the widespread adoption of these interventions in remote and home care settings.

## Introduction

Dysphagia is a swallowing disorder where foods or liquids fail to pass normally through the throat, often resulting in penetration or aspiration of food contents into the airway. In a survey of 4000 participants in the general population, more than 36% of respondents indicated dysphagia [1]. Furthermore, a systematic review reported a global dysphagia prevalence of 43.8% across diverse populations, noting an upward trend over time [2]. Dysphagia is common in high-risk groups, such as older adults and people with stroke, head and neck cancer, and neurodegenerative disease. An epidemiological survey conducted in China involving 5943 individuals indicated that the prevalence of dysphagia among healthy older adults, patients with stroke, head and neck cancer, and neurodegenerative disease was 19.2%, 51.1%, 34.4%, and 48.3%, respectively [3].

Dysphagia is associated with several complications, including an increased risk of malnutrition, aspiration, and pneumonia, leading to elevated medical expenses, prolonged hospitalizations, and greater mortality rates [3-5]. In addition, dysphagia has a direct negative effect on cognitive and psychosocial function. Several studies indicate that swallowing disorders are directly correlated with mild cognitive impairment, depression, anxiety, social isolation, and a reduced quality of life [6-9].

It is well-documented that swallowing exercises can improve the swallowing function, nutritional status, and quality of life of participants with swallowing disorders [10-12]. Generally, swallowing exercises require long-term persistence to achieve the expected results [13,14]. In addition, conventional swallowing exercises are mechanically repetitive and do not provide real-time feedback, resulting in a gradual decline in adherence [15,16]. Therefore, improving patient adherence is a key priority in swallowing rehabilitation.

Gamification involves applying game attributes in a nongaming context. In clinical rehabilitation, gamification utilizes game-like elements—such as point scoring, challenges, real-time feedback, and progression levels—to promote patient motivation, involvement, and adherence to therapy. In the context of dysphagia rehabilitation, these elements are embedded within exercises to make repetitive tasks more interactive and rewarding, potentially improving neuroplasticity and functional outcomes. For example, gamified swallowing exercises, coordinated with surface electromyography biofeedback (sEMG-BF), exhibited a greater improvement in the swallowing function of stroke patients than routine swallowing exercises or sEMG-BF alone [17]. Furthermore, the Park study demonstrated that gamified swallowing exercises could enhance training motivation, interest, enjoyment, and adherence in stroke patients [18].

Some researchers have applied gamified swallowing exercises in patients with swallowing disorders; however, the intervention effect of gamified swallowing exercises remains uncertain. For example, some studies have reported that gamified swallowing training can improve swallowing function to a greater extent than conventional swallowing training. In contrast, others have shown that its effects are similar to those of conventional swallowing training [17-19]. Although gamified swallowing exercises have gained attention, their effectiveness remains inconsistently reported, particularly regarding participant adherence, nutritional outcomes, and swallowing function. This inconsistency highlights a critical gap in the evidence base that this review seeks to address. Therefore, in this study, we aimed to evaluate how gamified swallowing exercises affect swallowing function, adherence, nutritional status, and quality of life in patients with dysphagia and advocate for their incorporation into clinical practice in swallowing rehabilitation.

## Methods

This systematic review and meta-analysis adhered to the PRISMA (Preferred Reporting Items for Systematic Reviews and Meta-Analyses) 2020 statement[20], as seen in . This study was registered in PROSPERO (International Prospective Register of Systematic Reviews) (CRD42024617169), and the protocol has been published [21]. Any deviations are detailed in Multimedia Appendix 1.

### Search Strategy

Seven English-language databases (PubMed, Web of Science, Embase, CINAHL, Cochrane Library, JBI, and Scopus) and 3 Chinese-language databases (China National Knowledge Infrastructure, SinoMed, and Wanfang) were systematically searched from inception to June 25, 2025. We conducted a repeated search on February 8, 2026, and found no more eligible literature. Google Scholar was retrieved as a gray literature to minimize publication bias. We retrieved the CENTRAL (Cochrane Central Register of Controlled Trials) database through the Cochrane Library, including resources from trial registries such as ClinicalTrials.gov and ChiCTR. No published search filters were used. The search terms “dysphagia,” “game,” and “swallowing exercise” were combined in each database using both free-text words and Medical Subject Heading terms, if available. We originally developed a scientific and rigorous search strategy based on our research question, under the guidance of a professor specializing in medical information retrieval. No previously published search strategies were adapted or reused in this review. The snowball method was used to retrieve references from eligible studies to identify potential literature and avoid missing studies. No language or other restrictions were applied to the search. The search strategy adhered to the PRISMA-S (Preferred Reporting Items for Systematic Reviews and Meta-Analyses Search Extension) checklist to ensure transparency [22] as seen inChecklist 2 . Multimedia Appendix 2 provides the specific search strategies for each database.

### Eligibility Criteria

The PICOS (population, intervention, comparison, outcomes, and study design) framework established the inclusion and exclusion criteria for the systematic review of randomized controlled trials (RCTs). The inclusion criteria were as follows: (1) population: patients with dysphagia aged 18 years or older, irrespective of the origin of the swallowing impairment; (2) intervention of interest: gamified swallowing exercises, defined as swallowing exercises carried out in the form of games, including video games, virtual reality games, etc; (3) comparison: individuals in the comparison group were provided with conventional swallowing exercises or nongamified exercises; (4) outcomes: swallowing function (including swallowing performance, dysphagia severity, and dysphagia screening), adherence, nutritional status, or quality of life; and (5) study design: RCTs (including crossover, cluster, and pilot studies). The exclusion criteria were as follows: (1) editorial articles, conference abstracts, study protocols, comments, and letters and (2) studies with essential data that remained unavailable if data conversion was not possible and the corresponding authors did not respond to our requests.

### Study Selection and Data Extraction

All retrieved studies were imported into EndNote 21 software for automatic and manual deduplication. After the removal of duplicates, 2 independent reviewers (JL and MY) screened the titles and abstracts before proceeding to assess the full texts against the established eligibility criteria. Disagreements during the study selection process were addressed through discussions within the research team.

Two reviewers (JL and MY) performed data extraction, with accuracy verification by a third researcher (MO). Information on the eligible studies was extracted, including study characteristics (authors, publication year, and country), participant characteristics (age and sample size), interventions (components, dose, timing, frequency, and length), controls, and outcomes. For studies with more than 1 intervention group where gamification was used in only 1 of the intervention groups, a comparison was conducted between the gamified group and the other 2 groups. If the authors did not report the mean and SD, we contacted the corresponding authors via email to request raw datasets. If unavailable, we converted them to the mean and SD based on the median and IQR [23,24]. Otherwise, the data were classified as missing data.

### Quality Appraisal

The revised Cochrane Risk of Bias Tool, version 2.0, was used to assess the methodological quality of the RCTs [25]. Five domains were assessed: randomization process, deviations from intended interventions, missing outcome data, measurement of the outcome, and selection of the reported result. The evaluation results were divided into low-risk, some concerns, and high-risk groups. Additionally, the GRADE (Grading of Recommendations, Assessment, Development, and Evaluation) framework was used to grade the certainty of evidence for all outcomes [26]. The certainty was divided into high, moderate, low, and very low based on the risk of bias, imprecision, inconsistency, indirectness, and publication bias [27]. The overall quality of evidence was evaluated using GRADEpro GDT software. Two reviewers (JL and MY) independently assessed the quality of the included studies. Disagreements were addressed through discussion and/or consultation with a third reviewer (MO) to achieve consensus.

### Data Statistics

Data were analyzed using R software (version 4.4.2). According to the Cochrane Handbook for Systematic Reviews of Interventions [28], for the 3-arm RCTs, we combined the 2 nongamified groups into 1 group, and pooled calculations were performed using the following formula:


$$N=N_{1}+N_{2}$$

$$M=\frac{N_{1}M_{1}+N_{2}M_{2}}{N_{1}+N_{2}}$$

$$SD=\sqrt{\frac{\left(N_{1}-1\right)SD_{1}^{2}+\left(N_{2}-1\right)SD_{2}^{2}+\frac{N_{1}N_{2}}{N_{1}+N_{2}}\left(M_{1}^{2}+M_{2}^{2}-2M_{1}M_{2}\right)}{N_{1}+N_{2}-1}}$$

A meta-analysis was conducted on outcomes measured in 2 or more studies. For continuous variables measured using the same scale, we estimated pooled effect sizes using the pooled mean difference (MD); otherwise, the pooled standardized mean difference (SMD) was computed as the pooled effect size. For studies that used multiple instruments to assess the same outcome, we established a priori “scale prioritization” rules based on the psychometric properties of each instrument (such as Gugging Swallowing Screen > Standardized Swallowing Assessment > Water Swallowing Test) [29], and extracted data accordingly for effect size synthesis. We employed the Hartung-Knapp-Sidik-Jonkman method for random-effects model analysis to calculate the pooled estimate and its corresponding 95% CI, addressing the issue of limited study inclusion and high heterogeneity between studies [30]. The heterogeneity of outcomes was tested using Cochran *Q* (*χ*^2^ test) and *I*^2^ statistic [31]. In detail, *P*<.05 was considered to indicate heterogeneity.

To explore potential sources of heterogeneity, subgroup analyses were conducted based on different assessment tools, kinds of games, different additional equipment, and disease duration. The Instrument for the Credibility of Effect Modification Analyses (ICEMAN) was applied to evaluate the credibility of subgroup effects [32] when the interaction *P*<.1. The credibility of subgroup effects was categorized as “very low,” “low,” “moderate,” or “high” based on 8 core evaluation items. An interaction *P*≤.005 was used as the threshold to minimize the likelihood of chance findings. Results for subgroups containing only a few studies were interpreted with caution.

Upon detecting substantial heterogeneity, 2 types of sensitivity analyses were conducted to evaluate the robustness of the findings as follows: (1) a leave-one-out method, where each study was sequentially excluded to assess changes in heterogeneity and result stability and (2) an alternative measurement instrument analysis, in which alternative measurement results were used for studies that assessed the same outcome using 2 different instruments. Funnel plots were used to assess small-study effects [33]. In addition, when the number of included studies was small, we calculated the 95% prediction interval using the Nagashima correction to illustrate the practical implications of heterogeneity and present the expected range of true effects across studies [34].

## Results

### Study Selection

**Figure 1.**
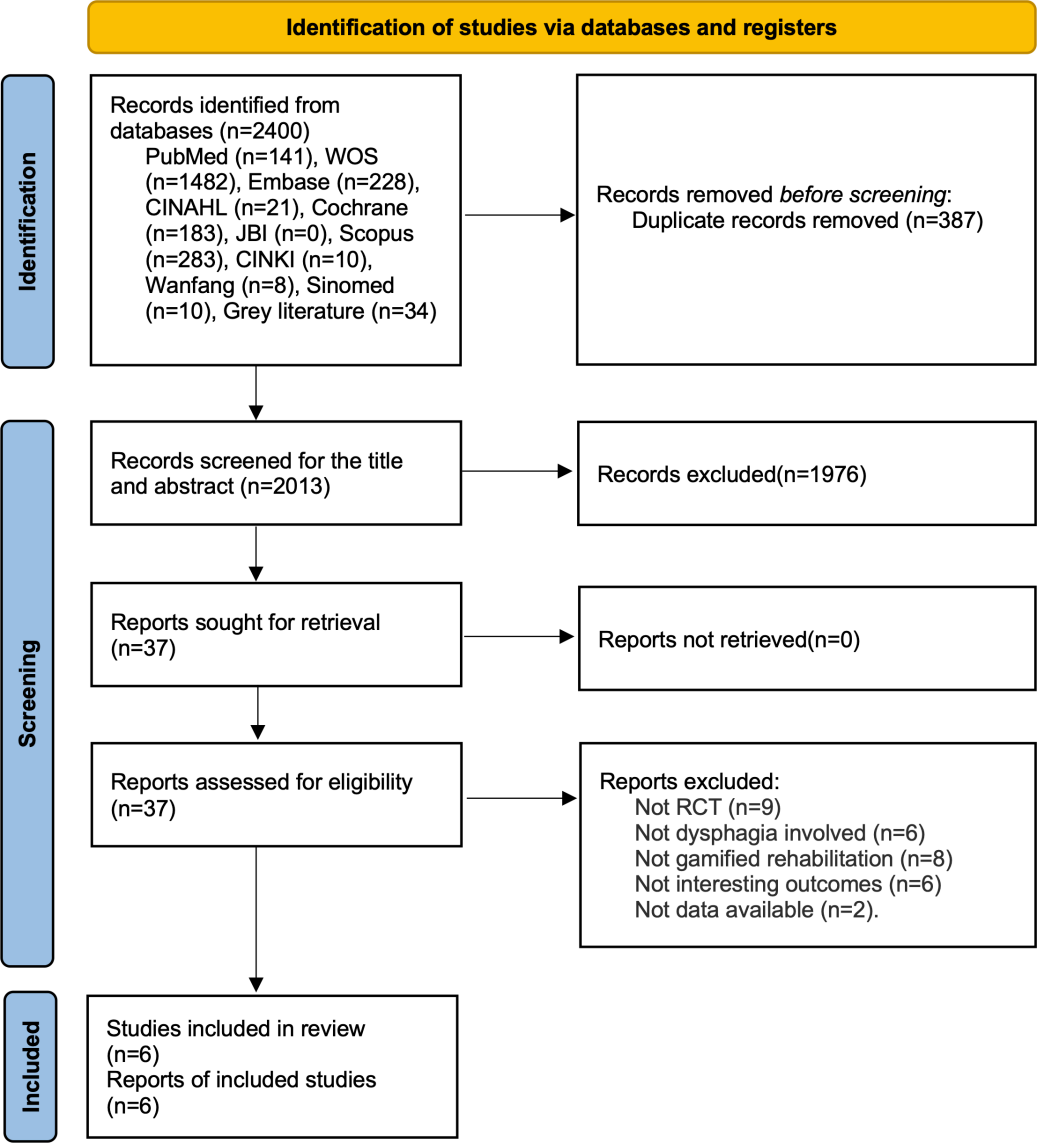
PRISMA (Preferred Reporting Items for Systematic Reviews and Meta-Analyses) flow diagram for study selection. RCT: randomized controlled trials.

The initial search identified 2400 publications, including 2366 records from 9 databases (the JBI database yielded no publications) and 34 from the gray literature database. After removing 387 duplicate articles, the titles and abstracts were scanned, and 1976 articles were excluded owing to irrelevant topics. After reviewing the full text, 31 articles were excluded because of their irrelevant study design, participants, interventions, outcomes, or unavailability of data. Ultimately, 6 studies [17-19,35-37] were included in this review. A flow diagram is presented in Figure 1, and the details of excluded studies are shown in Multimedia Appendix 3.

### Study Characteristics

Table 1 summarizes the characteristics of the 6 studies included in this review. All studies were published between 2019 and 2025. Four studies [17,19,36,37] were conducted in China, 1 [18] in Korea, and 1 [35] in Turkey. Five studies [18,19,35-37] were 2-arm RCTs, and 1 [17] was a 3-arm RCT. In total, the studies involved 330 participants with poststroke dysphagia. The disease duration ranged from 8 days (approximately 0.27 mo) to 5.18 months.

**Table 1.** Characteristics of included studies.

First author (y), country	Sample details (IG/CG^a^)	Diagnosis (IG/CG)	Intervention components (game name, details)	Equipment	Dose, frequency, and length of intervention	Control group	Measurements	Time points
Zhang (2025b) [36], China	Sample size: 42/42, age: 64.98 (SD 9.66)/66.43 (SD 13.12) y, male: 24/29, female: 18/13	Hemorrhagic stroke: 7/8, ischemic stroke: 35/34, disease duration: 1.67 (SD 1.48)/1.76 (SD 1.53) mo	AI^b^-based video game intervention: Lip exercise (“Collecting Carrots”: participants control the in-game character to move left and right by puffing out left or right cheeks (2‐3 s each time, 15 repetitions per cheek). When the game character reaches the target, carrots automatically drop into the backpack.) Tongue exercise (“Out of the Maze”: participants guide the game character through a maze by extending the tongue upwards, downwards, left, or right. Each tongue movement is repeated 15 times. The character exits the maze once the routine is completed.) CTAR^c^ exercise (“Leaping Barriers”: participants press a rubber ball with their chin, hold for 2 or 3 s, and then release, to make the bird descend and navigate around 15 obstacles in total.)	AI-based video game system and screen with an overhead camera	30 min per day, 5 times a week, for a total of 4 weeks	Usual care treatment, including lip exercises, tongue exercises, and CTAR	GUSS^d^, SSA^e^, FOIS^f^, MNA-SF^g^, SWAL-QOL^h^, and adherence	Pre/post, 4 wk follow-up
Park (2019) [18], South Korea	Sample size: 20/20, age: 60.95 (SD 11.19)/59.45 (SD 9.34) y, male:13/10, female:7/10)^i^	Hemorrhagic stroke: 12/14, ischemic stroke: 8/6, disease duration: 3.60 (SD 1.19)/3.85 (SD 1.18) mo	Game-based CTAR^c^: tucks the chin downward against a resistance bar to reach the target value displayed on the screen; 30 consecutive repetitions.	Tablet PC screen, a Bluetooth connector, or a resilient resistance bar	5 times a week, 4 wk	Head-lift exercise in the supine position	FOIS^f^, VDS^j^, and PAS^k^	Pre/post
Hou (2024) [17], China	Sample size: 30/60, age: 63.90 (SD 5.22)/63.88 (SD 6) y, male: 23/46, female: 7/14	Hemorrhagic stroke: 2/7, ischemic stroke: 28/53, disease duration: 8.60 (SD 3.50)/8.09 (SD 3.84) d	Game-based Mendelsohn maneuver (“Rabbit mountain-climbing game”: patient imagines themselves as a rabbit climbing a mountain. During the climbing process, they are instructed to perform the Mendelsohn maneuver and maintain it for a few seconds. After reaching the top of the mountain, the patient immediately exhales and relaxes, and the rabbit lies down and eats a carrot.) + usual care + tDCS^l^ + surface EMG-BF^m^	American VitalStim Plus 5923‐3 Electrotherapy System, computer; EMG-BF	20 min each time, once daily, 7‐14 d	Group 1: usual care + tDCS^l^, group 2: usual care + tDCS^l^ + sEMG-BF^m^	FOIS^f^, WST^n^, amplitude of the submental muscle group based on sEMG^J^, and tongue pressure	Pre/post
Zhang (2025a) [19], China	Sample size: 14/12, age: 66.50 (SD 8.25)/66.20 (SD 14.99) y, male: 8/10, female: 6/2	Hemorrhagic stroke: 3/2, ischemic: 11/10, disease duration: 1.91 (SD 0.85)/2.19 (SD 2.21) mo	Face recognition-driven video game swallowing training program: Lip exercises (“Carrot gathering”: participants control an in-game character to move left and right by alternately puffing out their left and right cheeks, maintaining each movement for 2‐3 s, 15 repetitions for each cheek. As the game character reaches designated spots, carrots automatically drop into the backpack.) Tongue exercises (“Labyrinth Navigation”: participants guide a character through a maze by extending the tongue upwards, downwards, left, or right, with 15 repetitions for each direction required to complete the maze.) Jaw exercises (“Soaring Bird”: participants control a bird descending and navigating around 15 obstacles by lowering their jaw to press a rubber ball with their chin for 2‐3 s, and then releasing.)	Face recognition–driven video game system, computer screen, and camera (recognize and record the participant’s facial movements)	30 min per day, 5 d per week, for 4 wk	Conventional therapy: exercises are identical to the intervention group, except without the FR-VG system	GUSS^d^, SSA^e^, FOIS^f^, SWAL-QOL^h^, VVST^o^, and adherence	Pre/post
Kang (2024) [37], China	Sample size: 30/30, age: 61.57 (SD 10.79)/63.40 (SD 10.26) y, male: 23/21, female: 7/9	Hemorrhagic stroke: 16/17, ischemic stroke: 14/13, disease duration: 32.03 (SD 8.72)/32.07 (SD 11.30) d	Virtual reality–based swallowing game: participant wears a head-mounted VR device, and when swallowing forcefully, the electromyographic signals of the sublingual muscles are transmitted to the VR device, triggering a VR scenario game. When the electromyographic signals exceed the set threshold, the virtual game character will successfully ingest food and earn points.	Pico 4 All-in-one VR Headset, computer; JP-001 sEMG Signals Acquisition System	30 min per day, 5 d per week, for 3 wk	Conventional therapy	SSA^e^, FOIS^f^, WST^n^, and surface electromyographic value	Pre/post
Alyanak (2025) [35], Turkey	Sample size: 16/17, age: 63.38 (SD 11.38)/60.59 (SD 12.54) y, male: 11/11, female: 5/6	Hemorrhagic stroke: 1/2, ischemic stroke: 15/15; disease duration: 5 (SD 2.84)/5.18 (SD 2.02) mo	Game-based EMG-BF^m^ therapy Effortful swallow (“Rose game”: patients press their tongue hard against the palate and swallow with all their strength; a rose on the computer screen would fade when muscle activity exceeds a threshold, and then, the muscles relax, and the rose blooms again.) Mendelsohn maneuver (“Rabbit game”: patients elevate the larynx to reach the highest point for 2‐3 s (Mendelsohn maneuver) to make the rabbit climb a hill and reach the top with the carrot, and then relax for 2 s, the rabbit would descend the hill and eat the carrot.) Each game is 15 min.	Vitalstim Plus device, the Vitalstim Software, computer; EMG-BF	30 min per day, 5 d per week, 3 wk	Effortful swallow + Mendelsohn maneuver with only verbal feedback	FOIS^f^, PAS^k^, DOSS^p^, and DHI^q^	Pre/post

a IG/CG: intervention group/control group.

b AI: artificial intelligence.

c CTAR: chin tuck against resistance.

d GUSS: Gugging Swallowing Screen.

e SSA: standardized swallowing assessment.

f FOIS: Functional Oral Intake Scale.

g MNA-SF: Mini Nutritional Assessment Short Form.

h SWAL-QOL: Swallowing Quality-of-Life Questionnaire.

i The study initially presented characteristics for 20 participants per group. However, the analysis was ultimately conducted on 37 participants (19 in the intervention group and 18 in the control group). We attempted to clarify this discrepancy with the corresponding author but received no response. Therefore, we proceeded to use the data from the 37 participants in our meta-analysis.

j VDS: Videofluoroscopic Dysphagia Scale.

k PAS: Penetration-Aspiration Scale.

l tDCS: transcranial direct current stimulation.

m sEMG-BF: surface electromyography biofeedback.

n WST: Water Swallowing Test.

o VVST: Volume-Viscosity Swallow Test.

p DOSS: Dysphagia Outcome and Severity Scale.

q DHI: Dysphagia Handicap Index.

### Intervention Characteristics

Table 1 summarizes the intervention characteristics of the 6 studies included in this review. The content and form of the gamified swallowing training varied. Three studies [17,18,37] included only 1 game module, and 3 studies [19,35,36] designed various games targeting specific swallowing movements. In terms of exercise content, 6 studies included tongue exercises (n=2), chin tuck against resistance exercises (n=2), lip movements (n=2), effortful swallowing (n=2), the Mendelsohn maneuver (n=2), and jaw exercises (n=1) [17-19,35-37].

All 6 studies required additional equipment (eg, surface electromyography, resistance bars, and computers equipped with face recognition–driven technology or artificial intelligence technology for intervention delivery) to conduct the interventions.

The gamified swallowing exercises in all studies were conducted for a period of 1 to 4 weeks [17-19,35-37], with only 1 study conducting a 4-week follow-up [36].

### Control Characteristics

Table 1 summarizes the characteristics of the control groups of the 6 included studies. Swallowing exercise protocols were identical between the control and intervention groups, except that gaming components were excluded in the controls. The usual care or conventional therapy typically encompassed oral motor exercises, sensory stimulation techniques, compensatory strategies, and supportive care (eg, vital sign monitoring, routine airway humidification, and nutritional management) [17-19,35-37].

### Outcomes

Swallowing function was the primary outcome in 6 studies [17-19,35-37]. Swallowing function was categorized into swallowing performance, dysphagia severity, and dysphagia screening based on scale items. Swallowing performance was primarily assessed through the Functional Oral Intake Scale (n=6) [17-19,35-37], which measures the patients’ ability to eat orally. The Dysphagia Outcome and Severity Scale (n=1) [35] and videofluoroscopic Dysphagia Scale (n=1) [18] were used to measure dysphagia severity. The measurement tools of dysphagia screening included the Gugging Swallowing Screen (GUSS; n=2) [19,36], the Standardized Swallowing Assessment (SSA; n=3) [19,36,37], and the Water Swallowing Test (WST; n=2) [17,37]. Physiological measures included submental muscle amplitude, tongue pressure, and surface electromyographic value [17,37].

### Risk of Bias

**Figure 2.**
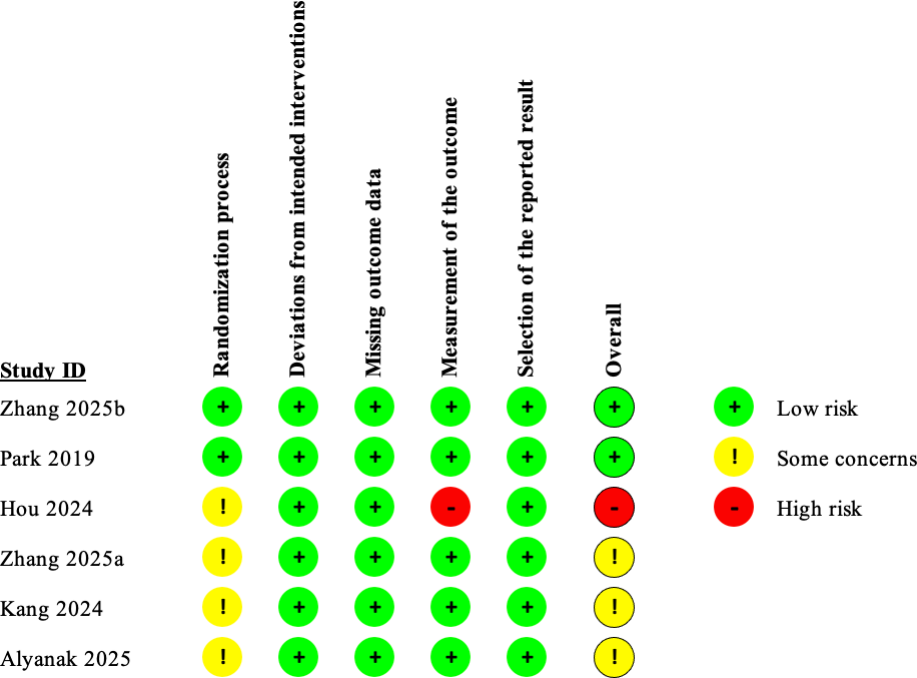
Risk-of-bias assessment of all included studies [17-19,35-37].

Figure 2 summarizes the risk of bias for the 6 included studies. Two reviewers independently assessed the methodological quality, achieving 95% agreement (κ=0.91). Two studies had a low risk of bias, 3 raised some concerns, and 1 was high risk. Three studies were considered to have some concerns because they did not report allocation concealment. One study had a high risk of bias because the outcomes were assessed without blinding, and the results, particularly those based on subjective measures, were therefore susceptible to influence by the outcome assessors. For a detailed assessment, please refer to Multimedia Appendix 4.

### Meta-Analysis

#### Effectiveness of Gamified Swallowing Exercises on Swallowing Function

##### Effectiveness of Gamified Swallowing Exercises on Swallowing Performance

**Figure 3.**
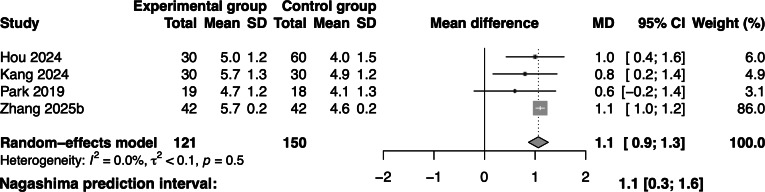
Forest plot of gamified swallowing exercises on swallowing performance [17,18,36,37].

Four studies involving 271 participants reported data on swallowing performance. The meta-analysis revealed a significant positive effect of gamified swallowing exercises on swallowing performance (MD=1.1; 95% CI 0.9‐1.3; *I*^2^=0; Figure 3) compared with the control group.

##### Effectiveness of Gamified Swallowing Exercises on Dysphagia Severity

**Figure 4.**
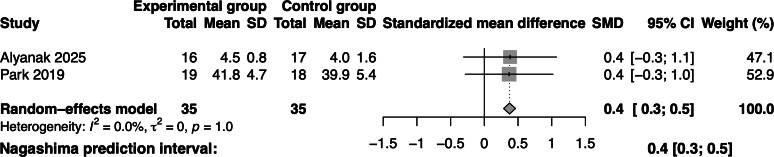
Forest plot of gamified swallowing exercises on dysphagia severity [18,35]. SMD: standardized mean difference.

A total of 2 studies involving 70 participants assessed dysphagia severity. Compared with the control group, the level of dysphagia severity in the gamified swallowing exercises group was significantly reduced (SMD=0.4; 95% CI 0.3‐0.5; *I*^2^=0; Figure 4).

##### Effectiveness of Gamified Swallowing Exercises on Dysphagia Screening

**Figure 5.**
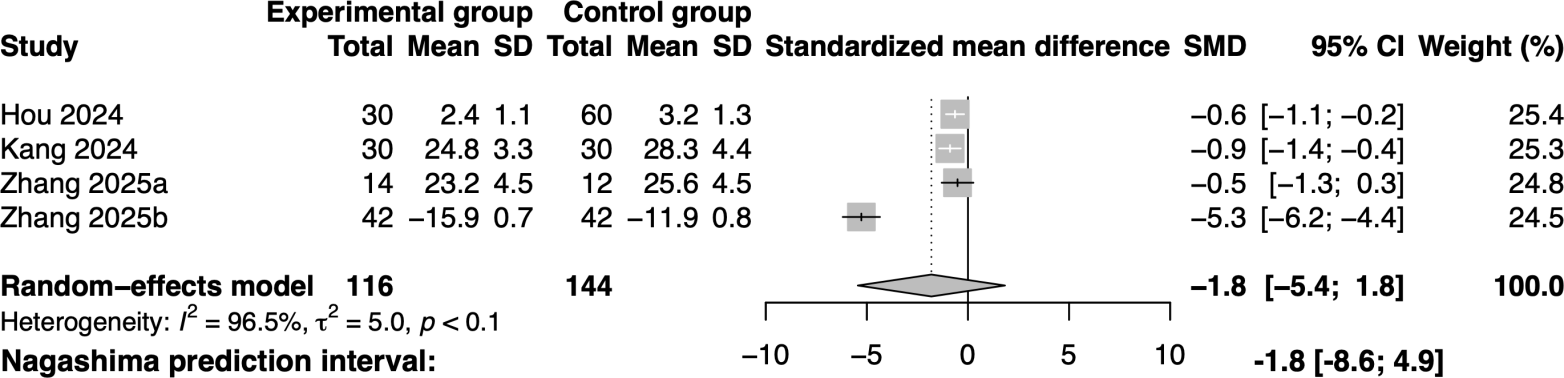
Forest plot of gamified swallowing exercises on dysphagia screening [17,19,36,37]. SMD: standardized mean difference.

The effectiveness of the gamified swallowing exercises on dysphagia screening was analyzed across 4 studies, including 260 participants. The meta-analysis demonstrated that the gamified swallowing exercises had no significant effect on dysphagia screening compared with the control group (SMD=–1.8; 95% CI –5.4 to 1.8; *I*^2^=96.5%; Figure 5).

### Effectiveness of Gamified Swallowing Exercises on Adherence

**Figure 6.**
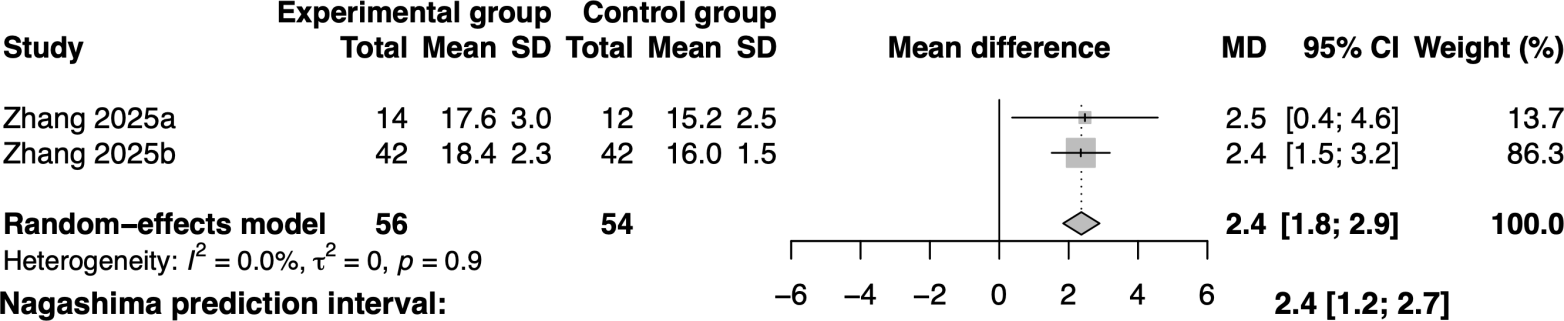
Forest plot of gamified swallowing exercises on adherence [19,36].

Regarding adherence, 2 studies involving 110 participants demonstrated that, compared with the control group, gamified swallowing exercises were associated with increased adherence among adults with dysphagia (MD=2.4; 95% CI 1.8‐2.9; *I*^2^=0; Figure 6).

### Effectiveness of Gamified Swallowing Exercises on Quality of Life

**Figure 7.**
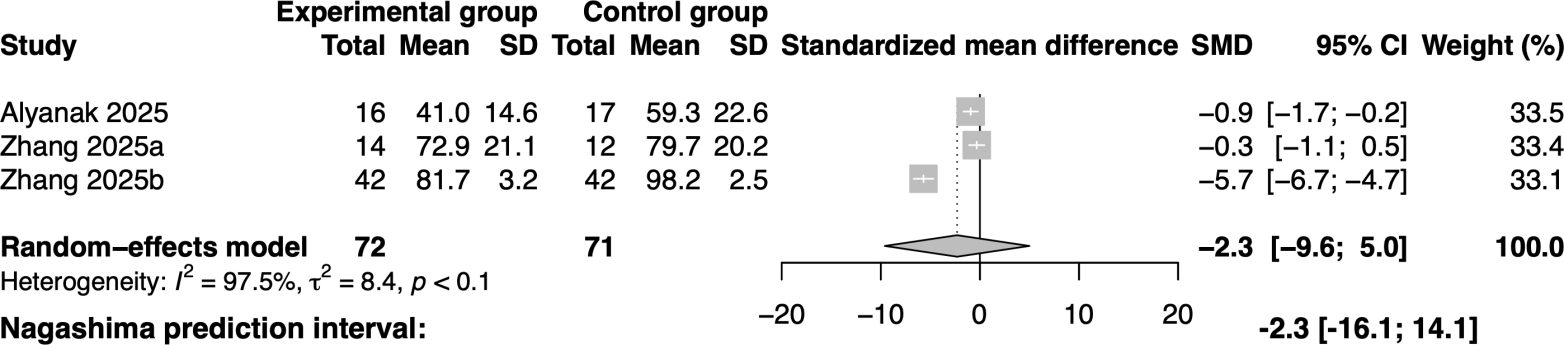
Forest plot of gamified swallowing exercises on quality of life [19,35,36]. SMD: standardized mean difference.

Quality of life was reported in 3 studies involving 143 participants. The results of the pooled analysis indicated that gamified swallowing training did not significantly improve the quality of life in adults with dysphagia compared with the control group (SMD=–2.3; 95% CI –9.6 to 5; *I*^2^=97.5%; Figure 7).

### Subgroup Analysis and Sensitivity Analysis

Given the preliminary findings of the meta-analysis, dysphagia screening was selected as the primary outcome for subgroup analysis. Subgroup analysis was conducted according to different measurement tools, kinds of games, additional equipment, and disease duration to explore the sources of heterogeneity. A significant between-subgroup difference was observed for the measurement tools (test for subgroup differences: *χ*²=86.5; 95% CI –5.4 to 1.8; *P*<.001). In contrast, no significant between-subgroup differences were found for kinds of games or additional equipment (*χ*²=0.8; 95% CI –5.4 to 1.8; *P*=.40) or disease duration (*χ*²=1; 95% CI –5.4 to 1.8; *P*=.30). The subgroup analysis showed that for patients with dysphagia after stroke, when using the SSA (k=2) to assess dysphagia screening, using a single game or using sEMG as the exercise equipment in dysphagia screening, the heterogeneity between studies significantly decreased (*I*^2^=0%), suggesting that such studies were relatively consistent in terms of design or implementation. However, the CI for the combined effect included the null value, indicating that the intervention effect was not statistically significant in this subgroup. Other subgroups, such as multiple games, other additional equipment (such as facial recognition devices), and disease duration ≥1 month, still had high heterogeneity (>95%). The remaining 3 subgroups (WST, GUSS, and disease duration <1 mo) had only 1 study, resulting in overall very low credibility of the findings, which should be interpreted with caution. Since the *P* value for the interaction effect in the kinds of games and additional equipment subgroup was >.1, it did not meet the prerequisites for applying the ICEMAN tool; therefore, no further evaluation was conducted. Detailed results of the subgroup analysis are illustrated in Multimedia Appendix 5.

To assess the impact of measurement tools on the robustness of the results, we performed an alternative measurement tool analysis, replacing the dysphagia screening scales used in Zhang study [36] and Kang study [37] with another scale. Specifically, we substituted GUSS with SSA in the Zhang study [36] and SSA with WST in the Kang study [37]. The direction of the pooled effect estimates remained unchanged after these substitutions. The leave-one-out sensitivity analysis indicated that the result of the swallowing performance was robust. For dysphagia screening, after excluding the Zhang study [36], the effect size of the remaining results changed (from ineffective to effective), and the heterogeneity decreased to 0, suggesting that the robustness for dysphagia screening was limited. For quality of life, if any 1 study was excluded, the effect size of the remaining results remained unchanged; however, after excluding the Zhang study [36], the heterogeneity significantly decreased (from 97.5% to 21.8%). Multimedia Appendix 6 presents the details of the sensitivity analysis.

However, due to the limited number of included studies, the estimation of the 95% prediction interval may be unstable, potentially leading to an overestimation of the true effects [38]. Therefore, the results of the prediction intervals should be interpreted with caution.

### Small-Study Effect Analysis

Due to the limited number of studies included in each funnel plot (n<8), the observed symmetry (or asymmetry) lacked reliability. Consequently, no meaningful assessment of funnel plot symmetry could be performed. The relevant funnel plots are provided in Multimedia Appendix 7.

### Nutritional Status

Among the included studies, only 1 [36] examined the effectiveness of gamified swallowing exercises on nutritional status, which precluded the ability to conduct a meta-analysis on nutritional status. The intervention group exhibited significantly higher nutritional scores compared with the control group (MD 1, SD 0.5, 95% CI 0.1-1.9; *P*=.03). However, the 4-week follow-up revealed no statistical significance in nutritional status between groups. These findings suggest that individuals with dysphagia who received gamified swallowing exercises achieved significantly better short-term nutritional status than those who received conventional swallowing exercises; however, further investigation is required to determine the long-term effects.

### Certainty of Evidence

Among the included outcomes, swallowing performance demonstrated moderate certainty of evidence; dysphagia severity and adherence had low certainty of evidence; and dysphagia screening, nutritional status, and quality of life showed very low certainty of evidence.

The certainty was downgraded primarily due to a high risk of bias, wide CIs, limited sample size, and statistical heterogeneity. Full GRADE (Grading of Recommendations Assessment, Development, and Evaluation) assessment details are available in Table 2.

**Table 2.** The details of the certainty of evidence.

Certainty assessment	No. of studies	Study design	Risk of bias	Inconsistency	Indirectness	Imprecision	Other considerations	Twin mix	Local anesthetic solution	Relative effect (95% CI)	Absolute effect (95% CI)	Certainty
Swallowing performance	4	Randomized trials	Serious^a^	Not serious	Not serious	Not serious	none	121	150	—^b^	MD 1.1 higher(0.9 higher to 1.3 higher)	⨁⨁⨁◯ Moderate^a^
Dysphagia severity	2	Randomized trials	Serious^a^	Not serious	Not serious	Serious^c^	None	35	35	—	SMD 0.4 higher (0.3 higher to 0.5 higher)	⨁⨁◯◯ Low^a^^c^
Dysphagia screening	4	Randomized trials	Serious^a^	Serious^d^	Not serious	Serious^e^	None	116	114	—	SMD 1.8 lower (5.4 lower to 1.8 higher)	⨁◯◯◯ Very low^a^^c^^e^
Adherence	2	Randomized trials	Not serious	Not serious	Not serious	Serious^f^	None	56	54	—	MD 2.4 higher (1.8 higher to 2.9 higher)	⨁⨁◯◯ Low^f^
Nutritional status	1	Randomized trials	Not serious^g^	Serious	Not serious	Serious^c^	None	42	42	—	MD 1 higher (1.9 lower to 0.1 lower)	⨁◯◯◯ Very low^c^^g^
Quality of life	3	Randomized trials	Serious^a^	Serious^d^	Not serious	Serious^c^	None	72	71	—	SMD 2.3 lower (9.6 lower to 5.0 higher)	⨁◯◯◯ Very low^a^^c^^d^

a Downgraded 1 level for serious risk of bias in the included studies.

b Not applicable.

c Downgraded 1 level for serious imprecision due to limited sample size.

d Downgraded 1 level for serious inconsistency due to statistical heterogeneity.

e Downgraded 1 level for serious imprecision due to wide CIs.

f Downgraded 2 levels for serious imprecision due to limited sample size and data from only 2 studies.

g Downgraded 1 level for serious inconsistency due to data from only 1 study, so it is impossible to judge the consistency.

## Discussion

### Principal Findings

Gamified swallowing exercise is an emerging and promising approach in the field of swallowing rehabilitation. To the best of our knowledge, this study is the first systematic review and meta-analysis to rigorously and comprehensively evaluate the effectiveness of gamified swallowing training. The findings suggest that gamified swallowing exercises may help improve swallowing performance, reduce dysphagia severity, and increase adherence among individuals with dysphagia. However, due to the limited number of studies and considerable heterogeneity, the effects of gamified swallowing training on dysphagia screening, nutritional status, and quality of life remain uncertain.

The meta-analysis indicated that gamified swallowing exercises can effectively enhance swallowing performance, reduce dysphagia severity, and improve adherence. Low heterogeneity and narrower prediction intervals also support higher consistency of the effects across different settings [39]. This positive outcome may be attributed to the integration of gamification elements into traditional swallowing exercises without altering the core components of the rehabilitation program. Compared to conventional approaches, the inherent mechanisms of gamification can stimulate participants to maintain their motivation, engagement, and interest, facilitating sustained participation in the process [40]. Moreover, immersive game-based interventions address key challenges in rehabilitation, such as monotony, participant discomfort, and resistance, which can foster positive attitudes toward swallowing exercises and promote long-term adherence to beneficial health behaviors [41].

Gamified swallowing rehabilitation systems typically employ additional equipment, such as EMG [17,35,37] or facial recognition devices [19,36], to monitor participants’ swallowing activity, including movements of the tongue, lips, jaw, and throat muscles. They then present the electrical activity of the relevant muscles or allow the participant to control a game character through these movements, providing real-time visual and auditory feedback. This equipment-assisted feedback mechanism helps participants promptly correct their training posture and reduces the workload of nurses and therapists [36]. Notably, the above equipment is specialized and can only be used in hospitals or rehabilitation institutions. Future development may focus on integrating this method with widely accessible mobile technologies, such as smartphones, to enable remote dysphagia management in community or home settings. As an innovative and scalable intervention model, gamified swallowing rehabilitation holds considerable promise for boosting engagement, promoting long-term recovery, and advancing the application of technology-assisted rehabilitation in real-world contexts.

Regarding dysphagia screening, this meta-analysis did not demonstrate a significant effect of gamified swallowing training, and wide prediction intervals indicate that the effect varies differently. During the subgroup analysis of dysphagia screening, the observation of zero heterogeneity in some subgroups points to consistent intervention design; however, the lack of significant therapeutic effect indicates that the intervention’s mechanism itself may need refinement. For example, swallowing is a multistage collaborative process, and a single game typically focuses on muscle training in one stage, which may limit the overall effectiveness of swallowing training. The limited number of studies in each subgroup reduced the statistical power, and the observed differences may reflect random error rather than true subgroup effects. These findings are exploratory and should be interpreted with caution, pending further validation through high-quality studies. Substantial statistical heterogeneity was observed within some subgroups (ie, multiple games, disease duration ≥1 mo), which may be attributable to the inclusion of a pilot study [19] with a small sample size, resulting in high uncertainty around its effect estimates. Furthermore, one included study [19] was a pilot version of another included trial [19,36]; however, these 2 studies exhibited markedly different standard deviations, with no overlap in confidence intervals, which further contributed to the heterogeneity.

The sensitivity analysis showed that removing the study by Zhang et al [36] would affect the direction of the results for dysphagia screening, indicating that, in the context of a limited number of studies, a single large-sample study might disproportionately impact the combined effect, reflecting the statistical vulnerability of meta-analyses with small samples rather than indicating a true difference in intervention effects.

Furthermore, several studies [17,19,37] had methodological limitations, primarily the lack of allocation concealment and blinding of outcome measurement. The former may introduce selection bias, whereas the latter can cause judgment bias in outcome assessments relying on subjective evaluations, which may lead to over- or under-estimation of effects and undermine the robustness of the combined results. These methodological flaws should be carefully considered when interpreting the research findings.

Overall, despite exploring multiple dimensions such as measurement tools, game quantity, additional equipment, and disease duration, the source of heterogeneity remains unclear. The findings of this study should be interpreted with caution, and future research should focus on large-scale RCTs while improving methodological quality and reporting standards to validate the intervention effects and clarify the sources of heterogeneity.

This meta-analysis revealed a potential positive impact of gamified swallowing exercises on reducing dysphagia severity. A major limitation, however, is the scarcity of available trials (n=2) and the small overall sample size (n=70). These constraints likely led to low statistical power, suggesting that the observed positive result should be considered preliminary and requires validation through larger, more robust studies. Furthermore, poststroke dysphagia often results from neurological impairment [42]. Due to the inclusion of participants who have had a stroke for >3 months, a longer intervention period may be required to achieve meaningful improvement [35].

Quality of life is a crucial long-term outcome indicator for assessing improvements in swallowing function [43]. The meta-analysis did not demonstrate a significant effect of gamified swallowing exercises on improving quality of life, and the wide prediction intervals indicate that the effects could vary widely, potentially benefiting or harming quality of life in different settings. Quality of life, a highly subjective and multidimensional outcome, is influenced by a range of factors, including psychological status, disease duration, and level of social support [44]. Furthermore, most included studies were constrained by small sample sizes and limited follow-up, with outcomes primarily assessed immediately post-intervention, which likely fails to capture the long-term efficacy of the swallowing exercises. Consequently, the current evidence is insufficient to draw definitive conclusions regarding the effect on quality of life, and these findings must be interpreted with caution. Further high-quality studies with larger samples and extended follow-up periods are warranted to validate these effects.

### Limitations

Several limitations should be noted in this study. First, a total of 11 databases were systematically searched, and the search was updated before submission. Ultimately, only 6 RCTs involving 330 participants were included. The limited number of studies and small sample size may restrict the comprehensiveness and generalizability of the findings. Nevertheless, rigorous and standardized methods were applied to ensure the methodological quality and transparency of this review. Second, only 2 studies strictly implemented randomization and blinding, whereas the methodological quality of the remaining 4 studies was suboptimal and requires improvement.

### Implications for Future Practice and Research

This study indicated that gamified swallowing exercises have a positive impact on swallowing function and adherence in adults with dysphagia, making them a promising rehabilitation approach for clinical practice. When designing and developing games for swallowing training, it is necessary to consider the cause of dysphagia and the accessibility of rehabilitation. Game-based exercises can be precisely designed to align with key rehabilitation goals. Accordingly, oral swallowing disorders may be addressed through games that enhance the strength of the lip, tongue, and jaw muscles, while pharyngeal disorders may be targeted with games designed to strengthen the throat muscles. The 6 included studies all used specialized and complex equipment, such as EMG and facial recognition devices, and, considering the long-term rehabilitation needs of patients, decreasing equipment dependence for gamified swallowing rehabilitation as much as possible is necessary so that individuals with swallowing disorders can engage in gamified swallowing exercises at home.

The current evidence base is characterized by single-center designs and an inherent high risk of performance bias due to the inability to blind participants and therapists. Moreover, the evidence is markedly limited by the absence of long-term outcome data, with only 1 study reporting a 4-week follow-up, which would bring a significant concern given that swallowing rehabilitation is a protracted process. It is therefore imperative that future research prioritizes large-scale, multicenter RCTs with extended follow-up periods to minimize confounding factors and provide higher-quality evidence on the effectiveness and long-term effects of gamified swallowing exercises, as well as to further explore the underlying effect mechanisms of gamified swallowing training. Furthermore, research should also be expanded to develop etiology-specific protocols, moving beyond the current narrow focus on poststroke dysphagia.

### Conclusion

This is the first systematic review and meta-analysis to evaluate and synthesize the effects of gamified swallowing exercises among adults with dysphagia. The findings demonstrated potential benefits of gamified swallowing exercises in improving swallowing performance, reducing dysphagia severity, and enhancing adherence among adults with dysphagia, while its effects on dysphagia screening, nutritional status, and quality of life warrant additional research to establish conclusive evidence. Given the limited number and small sample size of included studies, as well as the generally low quality of evidence, these results should be interpreted with caution. This study provides some reference and guidance for healthcare providers in the field of swallowing rehabilitation, such as integrating gamification elements, designing and developing etiology-specific games, and simplifying rehabilitation equipment. In the field of swallowing rehabilitation, helping adults with swallowing difficulties overcome technical barriers and improving the feasibility and scalability of home-based gamified swallowing rehabilitation might be a promising future research direction.

## Supplementary material

10.2196/82017 Multimedia Appendix 1 Deviations from the protocol.

10.2196/82017 Multimedia Appendix 2 Search strategy for each electronic database.

10.2196/82017 Multimedia Appendix 3 The details of excluded studies.

10.2196/82017 Multimedia Appendix 4 Risk of bias summary of all included studies.

10.2196/82017 Multimedia Appendix 5 Subgroup analysis.

10.2196/82017 Multimedia Appendix 6 Sensitivity analysis.

10.2196/82017 Multimedia Appendix 7 Small-study effect analysis.

10.2196/82017 Checklist 1 PRISMA checklist.

10.2196/82017 Checklist 2 PRISMA-S checklist.

## Abbreviations

CENTRAL: Cochrane Central Register of Controlled Trials
EMG: electromyography
GRADE: Grading of Recommendations, Assessment, Development, and Evaluation
GUSS: Gugging Swallowing Screen
ICEMAN: Instrument for the Credibility of Effect Modification Analyses
MD: mean difference
PI: prediction interval
PICOS: population, intervention, comparison, outcomes, and study design
PRISMA: Preferred Reporting Items for Systematic Reviews and Meta-Analyses
PRISMA-S: Preferred Reporting Items for Systematic Reviews and Meta-Analyses Search Extension
PROSPERO: International Prospective Register of Systematic Reviews
RCT: randomized controlled trial
sEMG: surface electromyography
SMD: standardized mean difference
SSA: Standardized Swallowing Assessment
WST: Water Swallowing Test
