# Supplementary material for: Effectiveness of Gamified Swallowing Exercises in Adults With Dysphagia: Systematic Review and Meta-Analysis of Randomized Controlled Trials
Source: JMIR Serious Games. 2026 Mar 26;14:e82017. doi: 10.2196/82017 (PMC13021111; doi:10.2196/82017)
Supplement: Multimedia Appendix 1 [file games-v14-e82017-s001.docx]

Appendix 1 Deviations from the protocol

**1. Search time**

In the study protocol, we planned to initiate the literature search on March 1, 2025. However, owing to adjustments in the submission timeline, a second repeated search was conducted on June 25, 2025.

**2. Analysis software**

In the original protocol, we intended to perform meta-analysis using RevMan software, which is the most widely used tool for this purpose. Given the small number of included studies and at the editor’s suggestion, we adopted the Hartung–Knapp–Sidik–Jonkman method. As this approach is not supported by RevMan, we instead conducted the meta-analysis using R software.

**3. Subgroup analysis**

As specified in the study protocol, subgroup analyses were originally planned based on delivery mode (e.g., in-person therapy vs. tele-rehabilitation) and implementation format (e.g., virtual reality‑based games vs. real‑world games). However, after completing the literature search, all included studies adopted in-person therapy and involved gamified swallowing training delivered via mobile devices. Accordingly, the subgroup analyses were revised to examine whether additional devices were used and the number of games employed.
