# Supplementary material for: Effectiveness of Gamified Swallowing Exercises in Adults With Dysphagia: Systematic Review and Meta-Analysis of Randomized Controlled Trials
Source: JMIR Serious Games. 2026 Mar 26;14:e82017. doi: 10.2196/82017 (PMC13021111; doi:10.2196/82017)
Supplement: Multimedia Appendix 2 [file games-v14-e82017-s002.docx]

**Appendix 1 Search strategy for each electronic database**

**Pubmed**

| #1 | “deglutition Disorders”[Mesh] OR deglutition Disorder*[Title/Abstract] OR dysphag*[Title/Abstract] OR deglut*[Title/Abstract] OR swallow*[Title/Abstract] OR swallowing disorder*[Title/Abstract] OR oropharyngeal dysphagia[Title/Abstract] OR esophageal dysphagia[Title/Abstract] OR swallowing difficult[Title/Abstract] OR swallowing impair*[Title/Abstract] OR swallowing dysfunction[Title/Abstract] |
| --- | --- |
| #2 | “Video games”[Mesh] OR “gamification”[Mesh] OR “game theory”[Mesh] OR “Mobile applications”[Mesh] OR game*[Title/Abstract] OR gamif*[Title/Abstract] OR gaming[Title/Abstract] OR gamefield[Title/Abstract] OR gameplay*[Title/Abstract] OR gameful*[Title/Abstract] OR gamelike[Title/Abstract] OR game theory[Title/Abstract] OR game element*[Title/Abstract] OR game based[Title/Abstract] OR gamification based[Title/Abstract] OR gaming based[Title/Abstract] OR game mechanic*[Title/Abstract] OR game feature*[Title/Abstract] OR video gam*[Title/Abstract] OR videogame element*[Title/Abstract] OR game component*[Title/Abstract] OR exergame*[Title/Abstract] OR multiplayer[Title/Abstract] OR player[Title/Abstract] OR playing[Title/Abstract] OR contest*[Title/Abstract] OR app-based[Title/Abstract] OR Mobile applications[Title/Abstract] OR application based[Title/Abstract] OR WeChat-based[Title/Abstract] |
| #3 | “exercise”[Mesh] OR “Therapeutics”[Mesh] OR “rehabilitation”[Mesh] OR exerci*[Title/Abstract] OR therap*[Title/Abstract] OR therapeutics[Title/Abstract] OR exercise therapy[Title/Abstract] OR train*[Title/Abstract] OR treatment*[Title/Abstract] OR rehabilit*[Title/Abstract] OR intervention*[Title/Abstract] OR movement*[Title/Abstract] OR motion*[Title/Abstract] OR recover*OR maneuver*[Title/Abstract] OR swallow* exercise[Title/Abstract] OR swallowing therapy[Title/Abstract] OR dysphagia rehabilitation[Title/Abstract] OR dysphagia management[Title/Abstract] OR swallowing rehabilitation[Title/Abstract] OR swallow training[Title/Abstract] OR swallowing intervention[Title/Abstract] OR shaker[Title/Abstract] OR strength*[Title/Abstract] OR tongue hold swallow[Title/Abstract] OR Mendelsohn maneuver[Title/Abstract] OR Masako maneuver[Title/Abstract] OR Chin tuck against resistance exercise[Title/Abstract] OR supraglottic swallow maneuver[Title/Abstract] OR super-supraglottic swallow maneuver[Title/Abstract] OR tongue strengthening exercises[Title/Abstract] OR Falsetto maneuver[Title/Abstract] OR pharyngeal squeeze maneuver[Title/Abstract] OR head lift exercise[Title/Abstract] |
| #4 | #1 AND #2 AND #3 |
| Results | 141 |

**Web of science**

| #1 | TS=(deglutition Disorder* OR dysphag* OR deglut* OR swallow* OR swallowing disorder* OR oropharyngeal dysphagia OR esophageal dysphagia OR swallowing difficult OR swallowing impair* OR swallowing dysfunction) |
| --- | --- |
| #2 | TS=(game* OR gamif* OR gaming OR gamefield OR gameplay* OR gameful* OR gamelike OR game theory OR game element* OR game based OR gamification based OR gaming based OR game mechanic* OR game feature* OR video gam* OR videogame element* OR game component* OR exergame* OR multiplayer OR player OR playing OR contest* OR mobile applications OR app-based OR application based OR Wechat-based) |
| #3 | TS=(exerci* OR therap* OR therapeutics OR exercise therapy OR train* OR treatment* OR rehabilit* OR intervention* OR movement* OR motion* OR recover*OR maneuver* OR swallow* exercise OR swallowing therapy OR dysphagia rehabilitation OR dysphagia management OR swallowing rehabilitation OR swallow training OR swallowing intervention OR shaker OR strength* OR tongue hold swallow OR Mendelsohn maneuver OR Masako maneuver OR Chin tuck against resistance exercise OR supraglottic swallow maneuver OR supersupraglottic swallow maneuver OR tongue strengthening exercises OR Falsetto maneuver OR pharyngeal squeeze maneuver OR head lift exercise) |
| #4 | #1 AND #2 AND #3 |
| Results | 1482 |

**CINAHL**

| #1 | SU=(deglutition Disorder* OR dysphag* OR deglut* OR swallow* OR swallowing disorder* OR oropharyngeal dysphagia OR esophageal dysphagia OR swallowing difficult OR swallowing impair* OR swallowing dysfunction) |
| --- | --- |
| #2 | SU=(game* OR gamif* OR gaming OR gamefield OR gameplay* OR gameful* OR gamelike OR game theory OR game element* OR game based OR gamification based OR gaming based OR game mechanic* OR game feature* OR video gam* OR videogame element* OR game component* OR exergame* OR multiplayer OR player OR playing OR contest* OR mobile applications OR app-based OR application based OR Wechat-based) |
| #3 | SU=(exerci* OR therap* OR therapeutics OR exercise therapy OR train* OR treatment* OR rehabilit* OR intervention* OR movement* OR motion* OR recover* OR maneuver* OR swallow* exercise OR swallowing therapy OR dysphagia rehabilitation OR dysphagia management OR swallowing rehabilitation OR swallow training OR swallowing intervention OR shaker OR strength* OR tongue hold swallow OR Mendelsohn maneuver OR Masako maneuver OR Chin tuck against resistance exercise OR supraglottic swallow maneuver OR supersupraglottic swallow maneuver OR tongue strengthening exercises OR Falsetto maneuver OR pharyngeal squeeze maneuver OR head lift exercise) |
| #4 | #1 AND #2 AND #3 |
| Results | 21 |

**The Cochrane library**

| #1 | MeSH descriptor: [Deglutition Disorders] explode all trees OR (deglutition Disorder* OR dysphag* OR deglut* OR swallow* OR swallowing disorder* OR oropharyngeal dysphagia OR esophageal dysphagia OR swallowing difficult OR swallowing impair* OR swallowing dysfunction):ti,ab,kw |
| --- | --- |
| #2 | MeSH descriptor: [Game Theory] explode all trees OR MeSH descriptor: [Video Games] explode all trees OR MeSH descriptor: [Gamification] explode all trees OR MeSH descriptor: [Mobile Applications] explode all trees OR (game* OR gamif* OR gaming OR gamefield OR gameplay* OR gameful* OR gamelike OR game theory OR game element* OR game based OR gamification based OR gaming based OR game mechanic* OR game feature* OR video gam* OR videogame element* OR game component* OR exergame* OR multiplayer OR player OR playing OR contest* OR mobile applications OR app-based OR application based OR Wechat-based):ti,ab,kw |
| #3 | MeSH descriptor: [Exercise] explode all trees OR MeSH descriptor: [Therapeutics] explode all trees OR MeSH descriptor: [Rehabilitation] explode all trees OR MeSH descriptor: [Motion] explode all trees OR MeSH descriptor: [Movement] explode all trees OR (exerci* OR therap* OR therapeutics OR exercise therapy OR train* OR treatment* OR rehabilit* OR intervention* OR movement* OR motion* OR recover*OR maneuver* OR swallow* exercise OR swallowing therapy OR dysphagia rehabilitation OR dysphagia management OR swallowing rehabilitation OR swallow training OR swallowing intervention OR shaker OR strength* OR tongue hold swallow OR Mendelsohn maneuver OR Masako maneuver OR Chin tuck against resistance exercise OR supraglottic swallow maneuver OR supersupraglottic swallow maneuver OR tongue strengthening exercises OR Falsetto maneuver OR pharyngeal squeeze maneuver OR head lift exercise):ti,ab,kw |
| #4 | #1 AND #2 AND #3 |
| Results | 183 |

**Embase**

| #1 | 'dysphagia'/exp OR 'aphagopraxia':ti,ab,kw OR 'deglutition difficulty':ti,ab,kw OR 'deglutition disorder':ti,ab,kw OR 'deglutition disorders':ti,ab,kw OR 'difficult deglutition':ti,ab,kw OR 'difficulty in swallowing':ti,ab,kw OR 'difficulty swallowing':ti,ab,kw OR 'disphagia':ti,ab,kw OR 'dysphagias':ti,ab,kw OR 'swallowing difficult':ti,ab,kw  OR 'swallowing difficultness':ti,ab,kw OR 'swallowing difficulty':ti,ab,kw OR 'swallowing disorder':ti,ab,kw OR 'dysphagia':ti,ab,kw |
| --- | --- |
| #2 | 'gamification'/exp OR 'game'/exp OR 'video game'/exp OR 'mobile applications'/exp OR ' healthcare software'/exp OR 'game model':ti,ab,kw OR 'games, experimental':ti,ab,kw OR 'model, game':ti,ab,kw OR 'game*':ti,ab,kw OR 'gamif*':ti,ab,kw OR 'gaming':ti,ab,kw OR 'gamefield':ti,ab,kw OR 'gameplay*':ti,ab,kw OR 'gameful*':ti,ab,kw OR 'gamelike':ti,ab,kw OR 'game theory':ti,ab,kw OR 'game element*':ti,ab,kw OR 'game based':ti,ab,kw OR 'gamification based':ti,ab,kw OR 'gaming based':ti,ab,kw OR 'game mechanic*':ti,ab,kw OR 'game feature*':ti,ab,kw OR 'video gam*':ti,ab,kw OR 'videogame element*':ti,ab,kw OR 'game component*':ti,ab,kw OR 'exergame*':ti,ab,kw OR 'multiplayer':ti,ab,kw OR 'player':ti,ab,kw OR 'playing':ti,ab,kw OR 'contest*':ti,ab,kw OR 'mobile application*':ti,ab,kw OR 'mobile app':ti,ab,kw OR ' healthcare software':ti,ab,kw |
| #3 | 'exercise'/exp OR 'therapy'/exp OR 'rehabilitation'/exp OR 'training'/exp OR 'motion'/exp OR 'movement (physiology)'/exp OR 'exerci*':ti,ab,kw OR 'therap*':ti,ab,kw OR 'therapeutics':ti,ab,kw OR 'exercise therapy':ti,ab,kw OR 'train*':ti,ab,kw OR 'treatment*':ti,ab,kw OR 'rehabilit*':ti,ab,kw OR 'intervention*':ti,ab,kw OR 'movement*':ti,ab,kw OR 'rehabilitative treatment':ti,ab,kw OR 'motion*':ti,ab,kw OR 'recover*':ti,ab,kw OR 'maneuver*':ti,ab,kw OR 'swallow* exercise':ti,ab,kw OR 'swallowing therapy':ti,ab,kw OR 'dysphagia rehabilitation':ti,ab,kw OR 'dysphagia management':ti,ab,kw OR 'swallowing rehabilitation':ti,ab,kw OR 'swallow training':ti,ab,kw OR 'swallowing intervention':ti,ab,kw OR 'shaker':ti,ab,kw OR 'strength*':ti,ab,kw OR 'tongue hold swallow':ti,ab,kw OR 'mendelsohn maneuver':ti,ab,kw OR 'masako maneuver':ti,ab,kw OR 'chin tuck against resistance exercise':ti,ab,kw OR 'supraglottic swallow maneuver':ti,ab,kw OR 'supersupraglottic swallow maneuver':ti,ab,kw OR 'tongue strengthening exercises':ti,ab,kw OR 'falsetto maneuver':ti,ab,kw OR 'pharyngeal squeeze maneuver':ti,ab,kw OR 'head lift exercise':ti,ab,kw |
| #4 | #1 AND #2 AND #3 |
|  | 228 |

**Scopus**

| #1 | TITLE-ABS-KEY (“deglutition Disorder*” OR “dysphag*” OR “deglut*” OR “swallow*” OR “swallowing disorder*” OR “oropharyngeal dysphagia” OR “esophageal dysphagia” OR “swallowing difficult” OR “swallowing impair*” OR “swallowing dysfunction”) |
| --- | --- |
| #2 | TITLE-ABS-KEY (“game*” OR “gamif*” OR “gaming” OR “gamefield” OR “gameplay*” OR “gameful*” OR “gamelike” OR “game theory” OR “game element*” OR “game based” OR “gamification based” OR “gaming based” OR “game mechanic*” OR “game feature*” OR “video gam*” OR “videogame element*” OR “game component*” OR “exergame*” OR “multiplayer” OR “player” OR “playing” OR “contest*” OR “mobile applications” OR “app-based” OR “application based” OR “Wechat-based”) |
| #3 | TITLE-ABS-KEY (“exerci*” OR “therap*” OR “therapeutics” OR “exercise therapy” OR “train*” OR “treatment*” OR “rehabilit*” OR “intervention*” OR “movement*” OR “motion*” OR “recover*” OR “maneuver*” OR “swallow* exercise” OR “swallowing therapy” OR “dysphagia rehabilitation” OR “dysphagia management” OR “swallowing rehabilitation” OR “swallow training” OR “swallowing intervention” OR “shaker” OR “strength*” OR “tongue hold swallow” OR “Mendelsohn maneuver” OR “Masako maneuver” OR “Chin tuck against resistance exercise” OR “supraglottic swallow maneuver” OR “supersupraglottic swallow maneuver” OR “tongue strengthening exercises” OR “Falsetto maneuver” OR “pharyngeal squeeze maneuver” OR “head lift exercise”) |
| #4 | #1 AND #2 AND #3 |
| Results | 283 |

**JBI**

| #1 | (deglutition Disorder or dysphagia or deglut or swallow or swallowing disorder* or oropharyngeal dysphagia or esophageal dysphagia or swallowing difficult or swallowing impair* or swallowing dysfunction).ab,kw,ti. |
| --- | --- |
| #2 | (game* or gamif* or gaming or gamefield or gameplay* or gameful* or gamelike or game theory or game element* or game based or gamification based or gaming based or game mechanic* or game feature* or video gam* or videogame element* or game component* or exergame* or multiplayer or player or playing or contest* or mobile applications or app-based or application based or Wechat-based).ab,kw,ti. |
| #3 | (exerci* or therap* or therapeutics or exercise therapy or train* or treatment* or rehabilit* or intervention* or movement* or motion* or recover* or maneuver* or swallow* exercise or swallowing therapy or dysphagia rehabilitation or dysphagia management or swallowing rehabilitation or swallow training or swallowing intervention or shaker or strength* or tongue hold swallow or Mendelsohn maneuver or Masako maneuver or Chin tuck against resistance exercise or supraglottic swallow maneuver or supersupraglottic swallow maneuver or tongue strengthening exercises or Falsetto maneuver or pharyngeal squeeze maneuver or head lift exercise).ab,kw,ti. |
| #4 | #1 AND #2 AND #3 |
| Results | 0 |

**CNKI**

| #1 | TKA=吞咽困难 + 吞咽障碍 + 吞咽功能 + 吞咽功能障碍 + 吞咽功能减退 + 吞咽功能受损 + 吞咽能力受损 + 吞咽异常 + 吞咽功能异常 + 进食障碍 + 咽下困难 + 咽下障碍 + 吞咽失常 + 进食困难 |
| --- | --- |
| #2 | TKA=游戏 + 游戏化 + 游戏模式 + 视频游戏 + 手机游戏 + 小程序游戏 + 微信游戏 + 游戏护理 |
| #3 | TKA=训练 + 康复 + 锻炼 + 运动 + 干预 + 治疗+ 管理 + 动作范围练习 + 张口训练 + 发音训练 + Masako训练 + 门德尔松吞咽法 + 用力吞咽法 + 舌抗阻训练 + 声门上吞咽训练 + 超声门上吞咽训练 + Shaker训练 + CTAR训练 |
| #4 | #1 AND #2 AND #3 |
| Results | 10 |

**WanFang**

| #1 | 题名或关键词:(吞咽困难 OR 吞咽障碍 OR 吞咽功能 OR 吞咽功能障碍 OR 吞咽功能减退 OR 吞咽功能受损 OR 吞咽能力受损 OR 吞咽异常 OR 吞咽功能异常 OR 进食障碍 OR 咽下困难 OR 咽下障碍 OR 吞咽失常 OR 进食困难) |
| --- | --- |
| #2 | 题名或关键词:(游戏 OR 游戏化 OR 游戏模式 OR 视频游戏 OR 手机游戏 OR 小程序游戏 OR 微信游戏 OR 游戏护理) |
| #3 | 题名或关键词:(训练 OR 康复 OR 锻炼 OR 运动 OR 干预 OR 治疗OR 管理 OR 动作范围练习 OR 张口训练 OR 发音训练 OR Masako训练 OR 门德尔松吞咽法 OR 用力吞咽法 OR 舌抗阻训练 OR 声门上吞咽训练 OR 超声门上吞咽训练 OR Shaker训练 OR CTAR训练) |
| #4 | #1 AND #2 AND #3 |
| Results | 8 |

**SinoMed**

| #1 | ( "吞咽困难"[常用字段:智能] OR "吞咽障碍"[常用字段:智能] OR "吞咽功能"[常用字段:智能] OR "吞咽功能障碍"[常用字段:智能] OR "吞咽功能减退"[常用字段:智能] OR "吞咽功能受损"[常用字段:智能] OR "吞咽能力受损"[常用字段:智能] OR "吞咽异常"[常用字段:智能] OR "吞咽功能异常"[常用字段:智能] OR "进食障碍"[常用字段:智能] OR "咽下困难"[常用字段:智能] OR "咽下障碍"[常用字段:智能] OR "吞咽失常"[常用字段:智能] OR "进食困难"[常用字段:智能]) |
| --- | --- |
| #2 | ( "游戏"[常用字段:智能] OR "游戏化"[常用字段:智能] OR "游戏模式"[常用字段:智能] OR "视频游戏"[常用字段:智能]) |
| #3 | ( "训练"[常用字段:智能] OR "康复"[常用字段:智能] OR "锻炼"[常用字段:智能] OR "运动"[常用字段:智能] OR "干预"[常用字段:智能] OR "治疗"[常用字段:智能] OR "管理"[常用字段:智能] OR "动作范围练习"[常用字段:智能] OR "张口训练"[常用字段:智能] OR "发音训练"[常用字段:智能] OR "Masako训练"[常用字段:智能] OR "门德尔松吞咽法"[常用字段:智能] OR "用力吞咽法"[常用字段:智能] OR "舌抗阻训练"[常用字段:智能] OR "声门上吞咽训练"[常用字段:智能] OR "超声门上吞咽训练"[常用字段:智能] OR "Shaker训练"[常用字段:智能] OR "CTAR训练"[常用字段:智能]) |
| #4 | #1 AND #2 AND #3 |
| Results | 10 |

**Google scholar**

| #1 | All field =(deglutition Disorder* OR dysphag* OR deglut* OR swallow* OR swallowing disorder* OR oropharyngeal dysphagia OR esophageal dysphagia OR swallowing difficult OR swallowing impair* OR swallowing dysfunction) |
| --- | --- |
| #2 | All field=(game* OR gamif* OR gaming OR gamefield OR gameplay* OR gameful* OR gamelike OR game theory OR game element* OR game based OR gamification based OR gaming based OR game mechanic* OR game feature* OR video gam* OR videogame element* OR game component* OR exergame* OR multiplayer OR player OR playing OR contest* OR mobile applications OR app-based OR application based OR Wechat-based) |
| #3 | All field=(exerci* OR therap* OR therapeutics OR exercise therapy OR train* OR treatment* OR rehabilit* OR intervention* OR movement* OR motion* OR recover* OR maneuver* OR swallow* exercise OR swallowing therapy OR dysphagia rehabilitation OR dysphagia management OR swallowing rehabilitation OR swallow training OR swallowing intervention OR shaker OR strength* OR tongue hold swallow OR Mendelsohn maneuver OR Masako maneuver OR Chin tuck against resistance exercise OR supraglottic swallow maneuver OR supersupraglottic swallow maneuver OR tongue strengthening exercises OR Falsetto maneuver OR pharyngeal squeeze maneuver OR head lift exercise) |
| #4 | #1 AND #2 AND #3 |
| Results | 34 |
