# Supplementary material for: Effectiveness of Gamified Swallowing Exercises in Adults With Dysphagia: Systematic Review and Meta-Analysis of Randomized Controlled Trials
Source: JMIR Serious Games. 2026 Mar 26;14:e82017. doi: 10.2196/82017 (PMC13021111; doi:10.2196/82017)
Supplement: Multimedia Appendix 3 [file games-v14-e82017-s003.docx]

**Appendix 2 The details of excluded studies**

| **Studies** | **Not randomized controlled trial** | **Not dysphagia adults** | **Not gamified swallowing exercise** | **Not interesting outcomes** | **Data was not available** |
| --- | --- | --- | --- | --- | --- |
| 1.Game changer epidemiological studies | √ |  |  |  |  |
| 2.An intensive neurorehabilitation programme with sEMG biofeedback to improve swallowing in idiopathic Parkinson's disease (IPD): A feasibility study | √ |  |  |  |  |
| 3.Interactive gaming for evaluating dysphagia in ICU patients | √ |  |  |  |  |
| 4.A novel virtual reality blowgun game for rehabilitation of older adults: A preliminary case series | √ |  |  |  |  |
| 5.Rehabilitation in patients with cerebellar ataxias | √ |  |  |  |  |
| 6.Swallowing Training Combined With Game-Based Biofeedback in Poststroke Dysphagia | √ |  |  |  |  |
| 7.Tongue motor training support system | √ |  |  |  |  |
| 8.Treatment of Dysphagia with Biofeedback and Functional Electrical Stimulation in a Patient with Wallenberg Syndrome: A Prospective Case Report | √ |  |  |  |  |
| 9.Based on A Gamified Mobile Platformto Construct an Intervention Program for Swallowing Exerciseof Radiotherapy-Related Head and Neck Cancer Patients (Chinese) | √ |  |  |  |  |
| 10.Effect of Oral Health Education Using a Mobile App (OHEMA) on the Oral Health and Swallowing-Related Quality of Life in Community-Based Integrated Care of the Elderly: A Randomized Clinical Trial |  | √ |  |  |  |
| 11.Effortful Swallow vs. Mendelsohn maneuver: strength or endurance training for older adults |  | √ |  |  |  |
| 12.A new method for tongue rehabilitation with computer games: Pilot study |  | √ |  |  |  |
| 13.Studies on the effects of playing games in groups to improve oral function |  | √ |  |  |  |
| 14.Tongue Muscle Training App for Middle-Aged and Older Adults Incorporating Flow-Based Gameplay: Design and Feasibility Pilot Study |  | √ |  |  |  |
| 15.Tongue-Controlled Computer Game: A New Approach for Rehabilitation of Tongue Motor Function |  | √ |  |  |  |
| 16.Application of Action Observation Therapy in Swallowing Rehabilitation: A Randomised Controlled Study |  |  | √ |  |  |
| 17.Biofeedback as an Adjunctive Treatment for Post-stroke Dysphagia: A Pilot-Randomized Controlled Trial |  |  | √ |  |  |
| 18.Combining immersive exergaming with physiotherapy in a specialized intensive Parkinson’s disease rehabilitation program: a randomized controlled trial |  |  | √ |  |  |
| 19.Electromyography- and Bioimpedance-Based Detection of Swallow Onset for the Control of Dysphagia Treatment |  |  | √ |  |  |
| 20.Head and Neck Virtual Coach: A Randomized Control Trial of Mobile Health as an Adjunct to Swallowing Therapy During Head and Neck Radiation |  |  | √ |  |  |
| 21.Implementation of a Home-Based mHealth App Intervention Program With Human Mediation for Swallowing Tongue Pressure Strengthening Exercises in Older Adults: Longitudinal Observational Study |  |  | √ |  |  |
| 22.Interactive website for head and neck cancer patients: Adherence and coping program to prevent dysphagia after radiation |  |  | √ |  |  |
| 23.Effect of situational interactive respiratory training system on patients with respiratory dysfunction after stroke (Chinese) |  |  | √ |  |  |
| 24.Applicability and experiences of silverfit rephagia by patients with Huntington's disease in day care |  |  |  | √ |  |
| 25.Application of an application connected to a game with stimulation of mastication and swallowing muscle activity in elderly people with Parkinson's disease |  |  |  | √ |  |
| 26.Development of a Virtual Game with Android Operating System for the Rehabilitation of Chewing and Swallowing in the Elderly |  |  |  | √ |  |
| 27.Immersive Virtual Reality for Dysphagia Treatment After Stroke |  |  |  | √ |  |
| 28.Kangaroo Stimulation Game in Tracheostomized Intensive Care–Related Dysphagia: Interventional Feasibility Study |  |  |  | √ |  |
| 29.The Swallowing Intelligent Assessment System Based on Tongue Strength and Surface EMG |  |  |  | √ |  |
| 30.Increasing Adherence to Prophylactic Swallowing Exercises During Head and Neck Radiotherapy: The Multicenter, Randomized Controlled PRESTO-Trial |  |  |  |  | √ |
| 31.Effects of game-based surface electromyograhic biofeedback training on dysphagia caused by brainstem injury (Chinese) |  |  |  |  | √ |
