# Supplementary material for: Effectiveness of Gamified Swallowing Exercises in Adults With Dysphagia: Systematic Review and Meta-Analysis of Randomized Controlled Trials
Source: JMIR Serious Games. 2026 Mar 26;14:e82017. doi: 10.2196/82017 (PMC13021111; doi:10.2196/82017)
Supplement: Multimedia Appendix 4 [file games-v14-e82017-s004.docx]

**Appendix 4 Risk of Bias Summary of All Included Studies**

| Study ID | Domain 1. Randomization process | | | | Domain 2. Deviations from intended interventions | | | | | | | | Domain 3. Missing outcome data | | | | |
| --- | --- | --- | --- | --- | --- | --- | --- | --- | --- | --- | --- | --- | --- | --- | --- | --- | --- |
|  | 1.1 | 1.2 | 1.3 | Judgement | 2.1 | 2.2 | 2.3 | 2.4 | 2.5 | 2.6 | 2.7 | Judgement | 3.1 | 3.2 | 3.3 | 3.4 | Judgement |
| Zhang et al., 2025b[1] | Y | Y | N | Low | PY | PY | PN | NA | NA | Y | NA | Low | Y | NA | NA | NA | Low |
| Park et al., 2019[2] | Y | Y | N | Low | PY | PY | PN | NA | NA | Y | NA | Low | Y | NA | NA | NA | Low |
| Hou et al., 2024[3] | Y | NI | N | Some concerns | PY | PY | PN | NA | NA | Y | NA | Low | Y | NA | NA | NA | Low |
| Zhang et al., 2025a[4] | Y | NI | N | Some concerns | PY | PY | PN | NA | NA | Y | NA | Low | Y | NA | NA | NA | Low |
| Kang, 2024[5] | Y | NI | N | Some concerns | PY | PY | PN | NA | NA | Y | NA | Low | Y | NA | NA | NA | Low |
| Alyanak et al., 2025[6] | Y | NI | N | Some concerns | PY | PY | PN | NA | NA | Y | NA | Low | Y | NA | NA | NA | Low |

Continued

| Study ID | Domain 4. Measurement of the outcome | | | | | | Domain 5. Selection of the reported result | | | | Overall |
| --- | --- | --- | --- | --- | --- | --- | --- | --- | --- | --- | --- |
|  | 4.1 | 4.2 | 4.3 | 4.4 | 4.5 | Judgement | 5.1 | 5.2 | 5.3 | Judgement |  |
| Zhang et al., 2025b[1] | N | N | N | NA | NA | Low | Y | N | N | Low | Low |
| Park et al., 2019[2] | N | N | N | NA | NA | Low | Y | N | N | Low | Low |
| Hou et al., 2024[3] | N | N | NI | PY | PY | High | Y | N | N | Low | High |
| Zhang et al., 2025a[4] | N | N | N | NA | NA | Low | Y | N | N | Low | Some concerns |
| Kang, 2024[5] | N | N | N | NA | NA | Low | Y | N | N | Low | Some concerns |
| Alyanak et al., 2025[6] | N | N | N | NA | NA | Low | Y | N | N | Low | Some concerns |
