# Supplementary material for: Effectiveness of Gamified Swallowing Exercises in Adults With Dysphagia: Systematic Review and Meta-Analysis of Randomized Controlled Trials
Source: JMIR Serious Games. 2026 Mar 26;14:e82017. doi: 10.2196/82017 (PMC13021111; doi:10.2196/82017)
Supplement: Multimedia Appendix 5 [file games-v14-e82017-s005.docx]

**Appendix 5 Subgroup analysis**

**
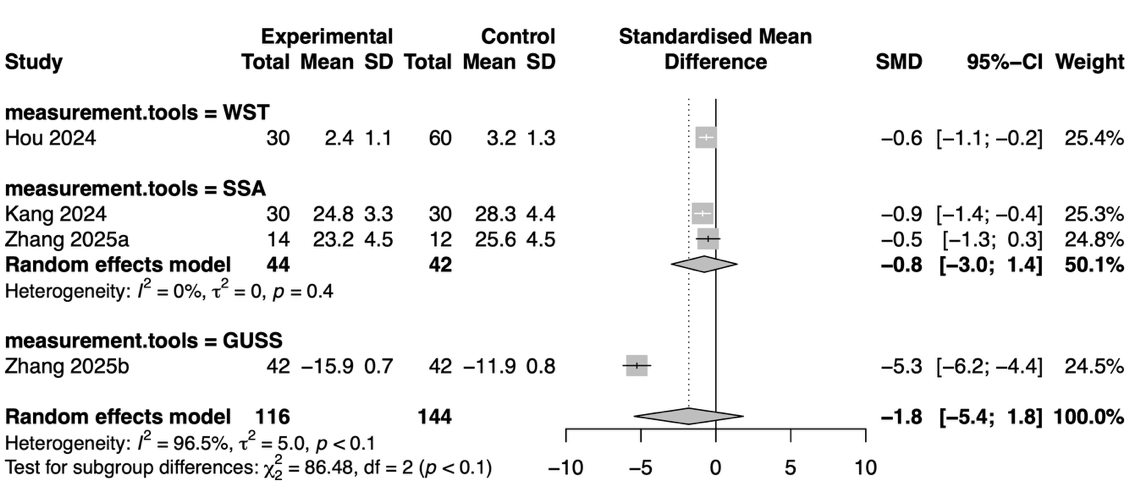
**

**Figure 1.** Forest plot: Effectiveness of gamified swallowing exercises with different measurement tools on the dysphagia screening


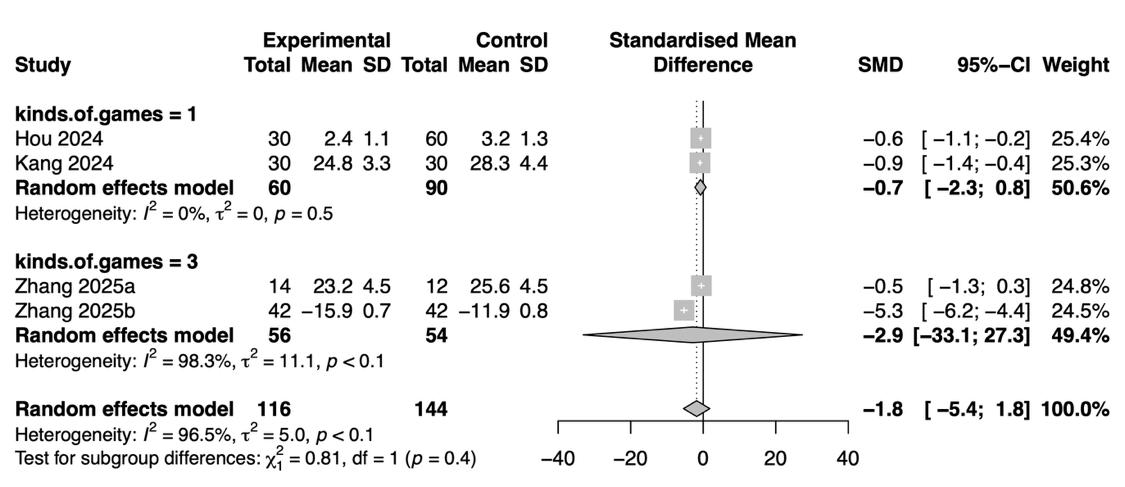


**Figure 2.** Forest plot: Effectiveness of gamified swallowing exercises with different kinds of games on the dysphagia screening


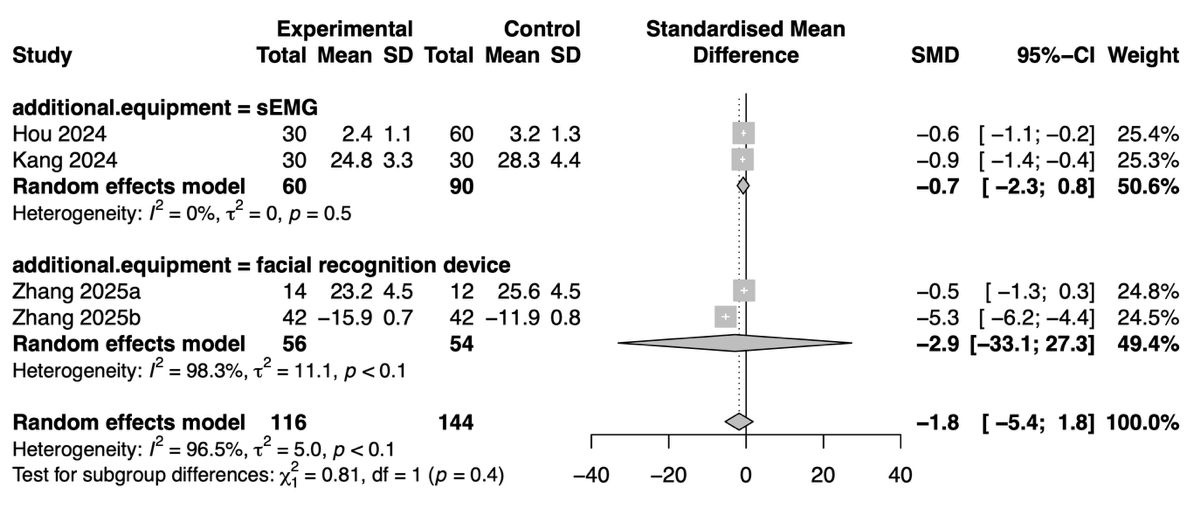


**Figure 3.** Forest plot: Effectiveness of gamified swallowing exercises with different additional equipment on the dysphagia screening


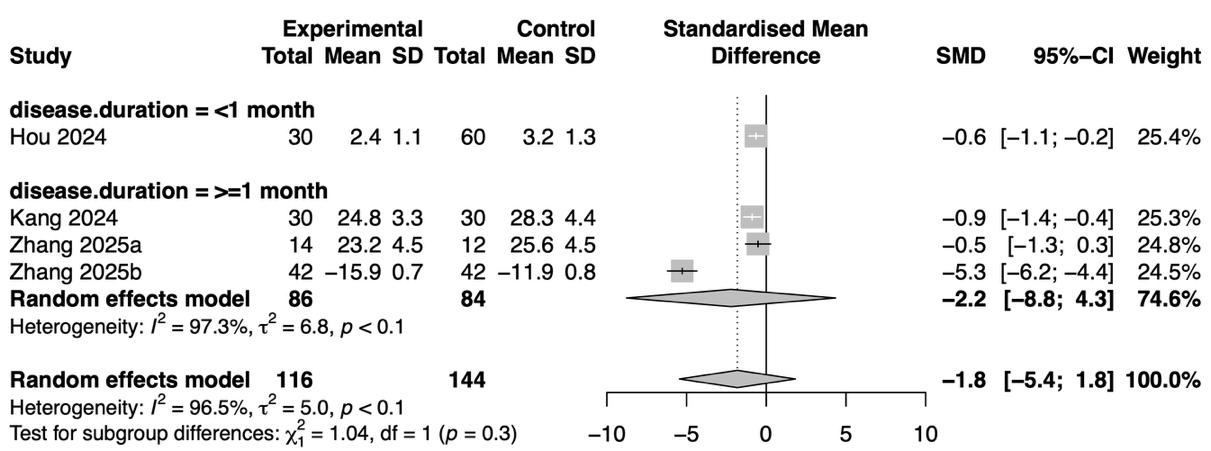


**Figure 4.** Forest plot: Effectiveness of gamified swallowing exercises with different disease duration on the dysphagia screening
