# Supplementary material for: Effectiveness of Gamified Swallowing Exercises in Adults With Dysphagia: Systematic Review and Meta-Analysis of Randomized Controlled Trials
Source: JMIR Serious Games. 2026 Mar 26;14:e82017. doi: 10.2196/82017 (PMC13021111; doi:10.2196/82017)
Supplement: Multimedia Appendix 6 [file games-v14-e82017-s006.docx]

**Appendix 6 Sensitivity analysis**

**1. Alternative measurement tool analysis
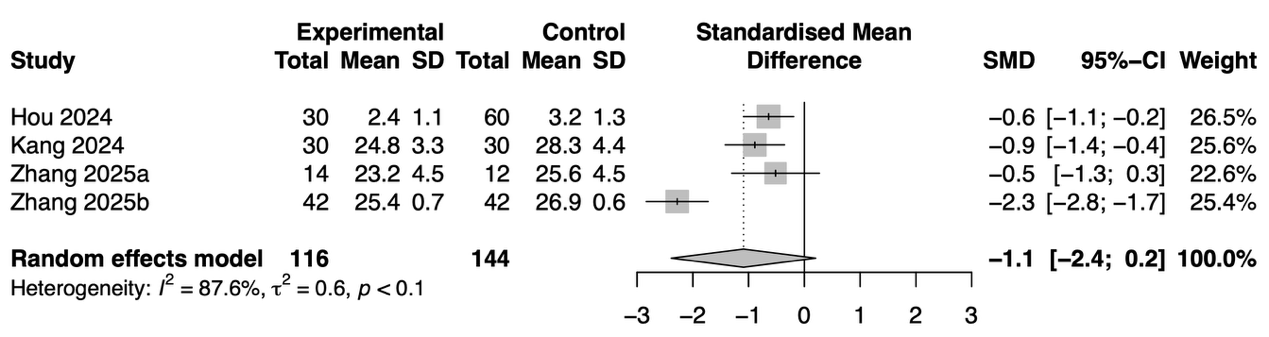
**

**Figure 1.** Forest plot: substitute Zhang(b)’s GUSS data with Zhang(b)’s SSA data

**
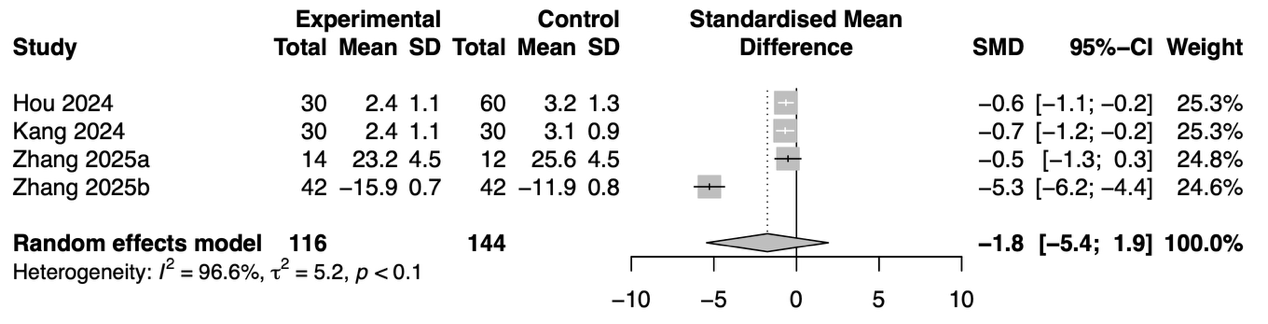
**

**Figure 2.** Forest plot: substitute Kang’s SSA data with Kang’s WST data

**
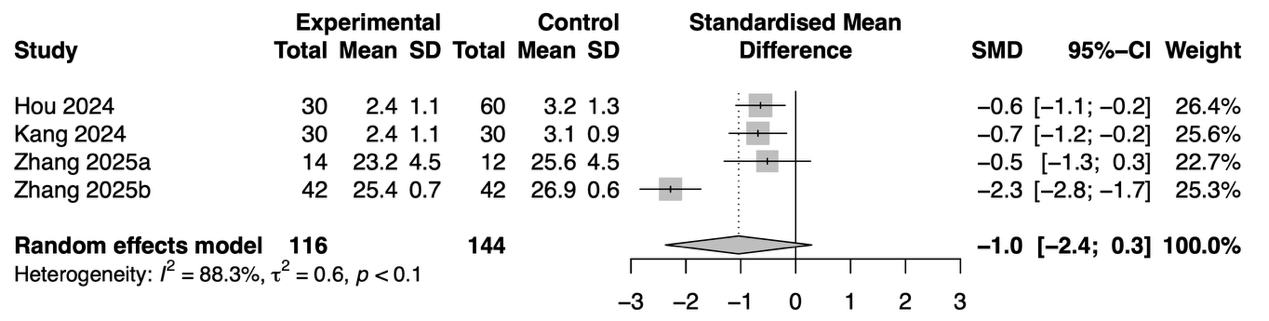
**

**Figure 3.** Forest plot: substitute Zhang(b)’s GUSS data with Zhang(b)’s SSA data and Kang’s SSA data with Kang’s WST data

**2. Leave-one-out sensitivity analysis**


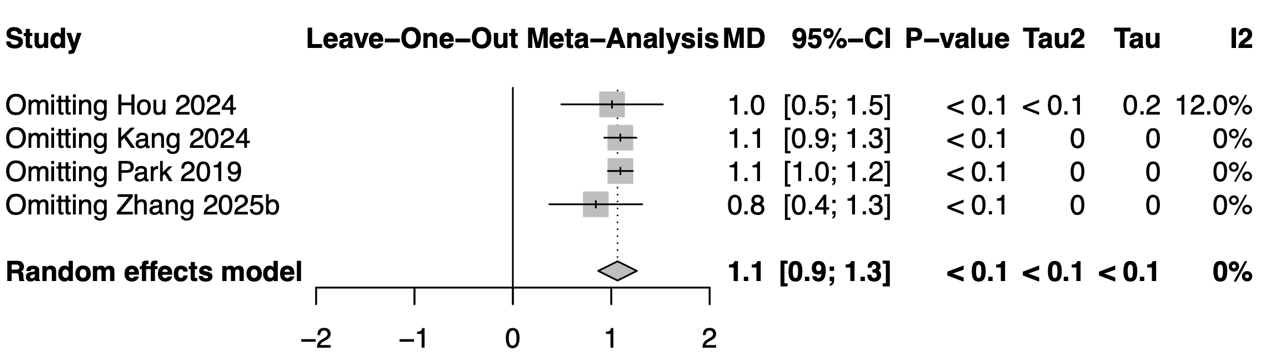


**Figure 4.** Leave-one-out sensitivity analysis of swallowing performance


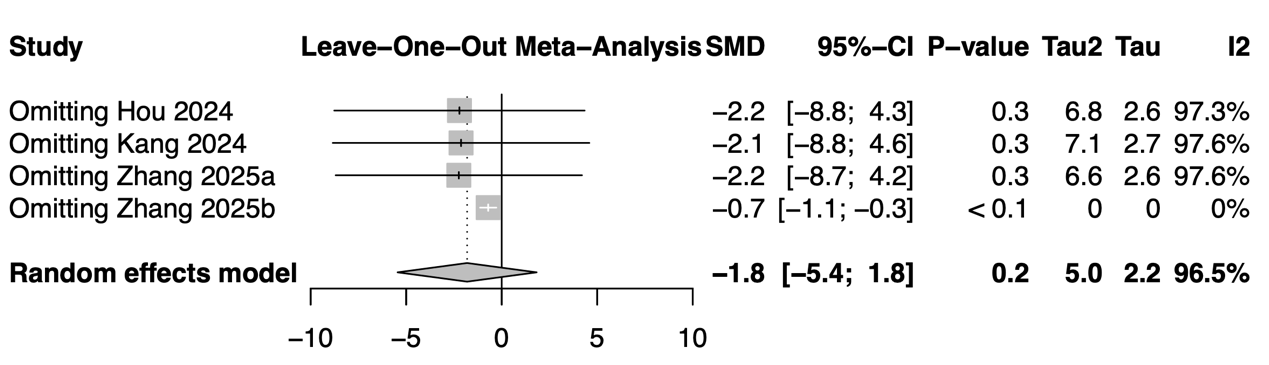


**Figure 5.** Leave-one-out sensitivity analysis of dysphagia screening


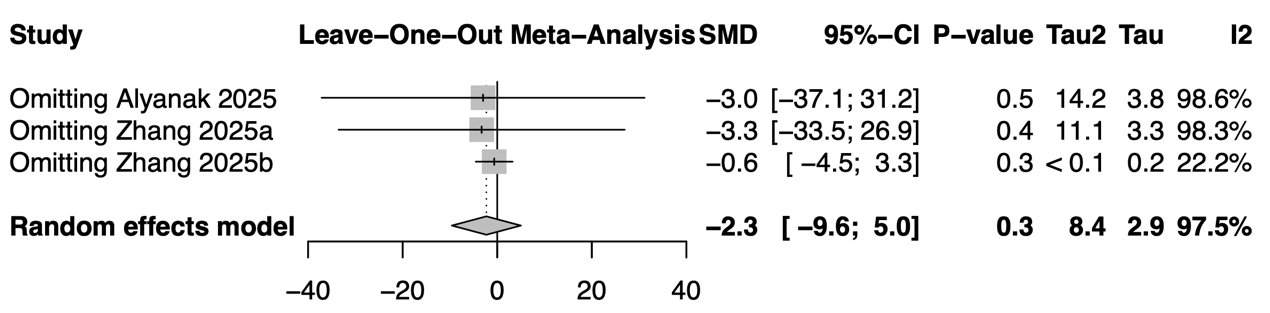


**Figure** **6.** Leave-one-out sensitivity analysis of quality of life
