# Supplementary material for: Effectiveness of Gamified Swallowing Exercises in Adults With Dysphagia: Systematic Review and Meta-Analysis of Randomized Controlled Trials
Source: JMIR Serious Games. 2026 Mar 26;14:e82017. doi: 10.2196/82017 (PMC13021111; doi:10.2196/82017)
Supplement: Multimedia Appendix 7 [file games-v14-e82017-s007.docx]

**Appendix 7 Small-study effect analysis**

**
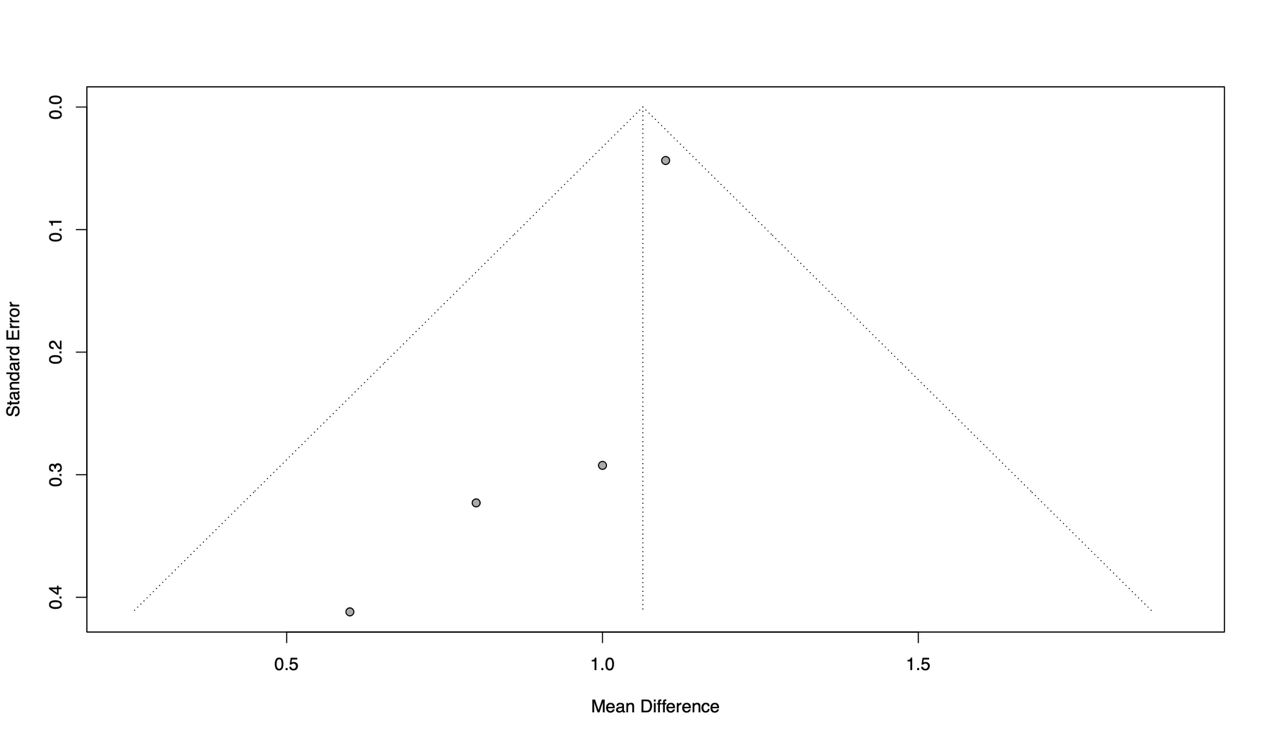
**

**Figure 1.** Funnel plot of swallowing performance


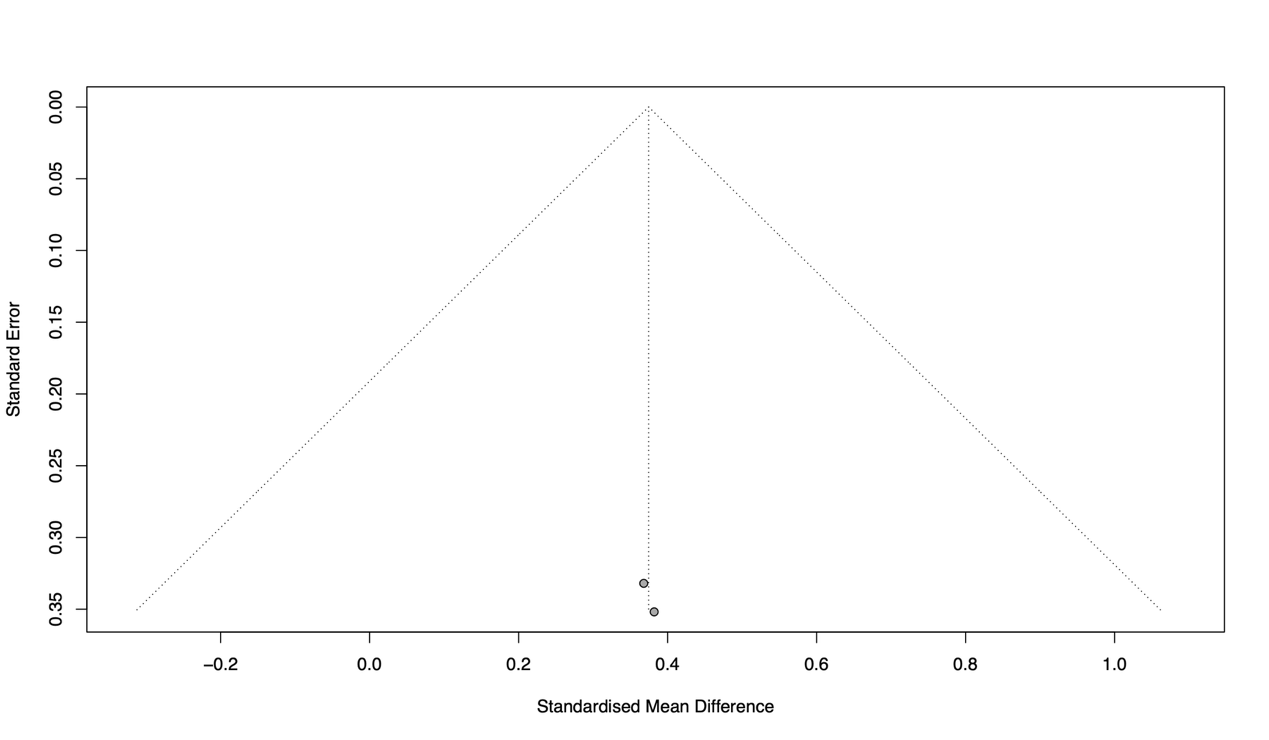


**Figure 2.** Funnel plot of dysphagia severity


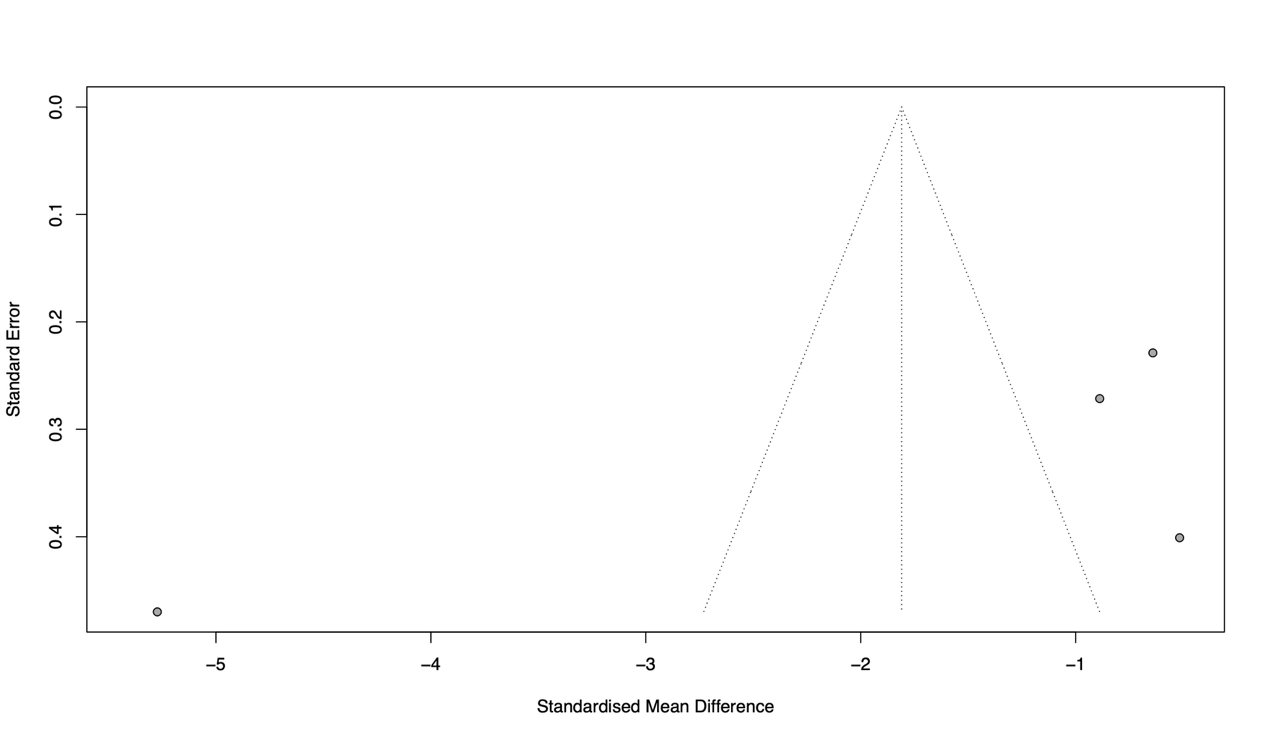


**Figure 3.** Funnel plot of dysphagia screening


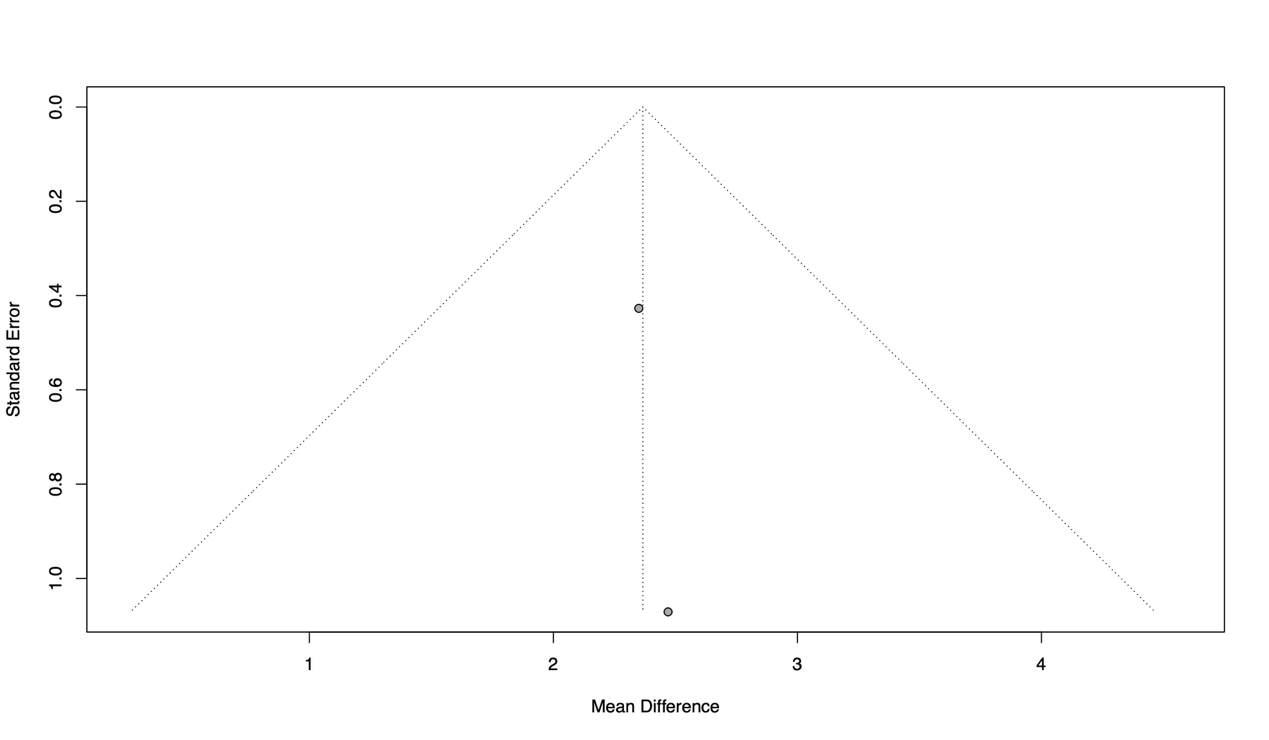


**Figure 4.** Funnel plot of adherence

**
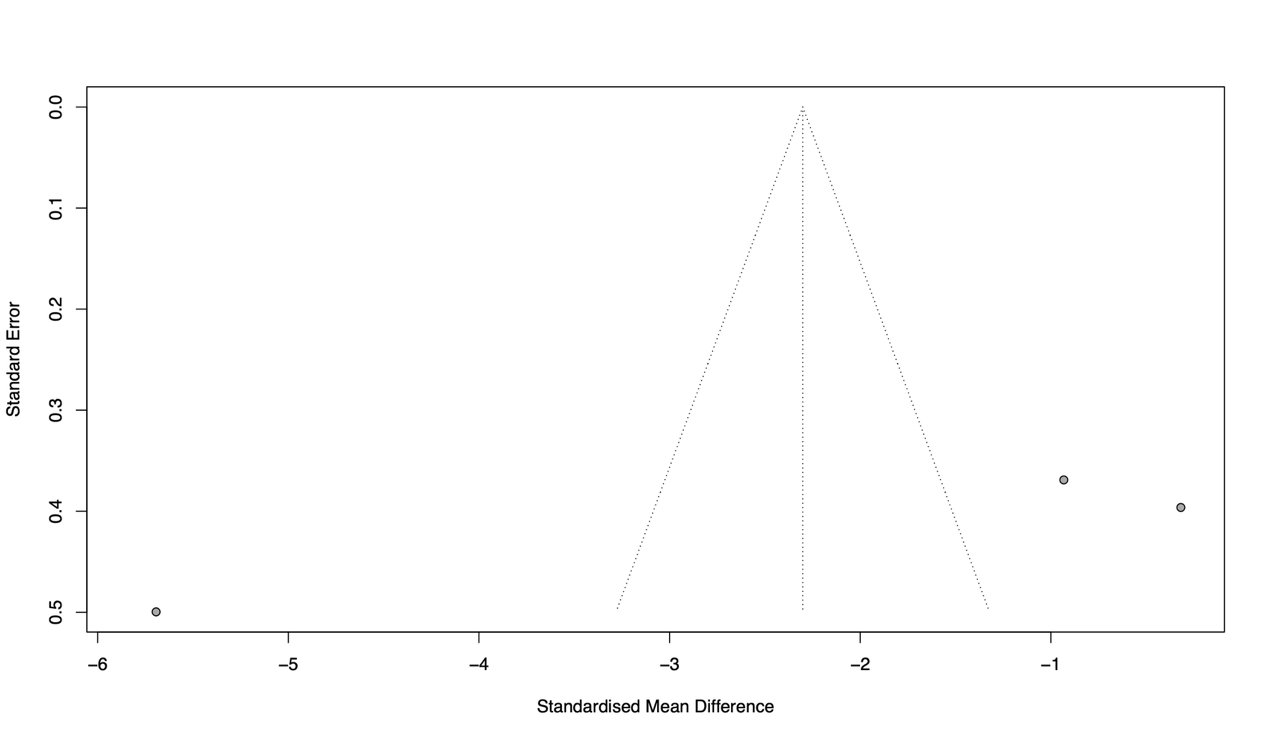
**

**Figure 5.** Funnel plot of quality of life
