# Supplementary material for: Effectiveness of Gamified Swallowing Exercises in Adults With Dysphagia: Systematic Review and Meta-Analysis of Randomized Controlled Trials
Source: JMIR Serious Games. 2026 Mar 26;14:e82017. doi: 10.2196/82017 (PMC13021111; doi:10.2196/82017)
Supplement: Checklist 2 [file games-v14-e82017-s009.pdf]

PRISMA-S Checklist

Section/topic	#	Checklist item	Location(s) Reported
**INFORMATION SOURCES AND METHODS**			
Database name	1	Name each individual database searched, stating the platform for each.	P4 Line 108-111
Multi-database searching	2	If databases were searched simultaneously on a single platform, state the name of the platform, listing all of the databases searched.	P5 Line 113-115
Study registries	3	List any study registries searched.	P5 Line 113-115
Online resources and browsing	4	Describe any online or print source purposefully searched or browsed (e.g., tables of contents, print conference proceedings, web sites), and how this was done.	P5 Line 112-113
Citation searching	5	Indicate whether cited references or citing references were examined, and describe any methods used for locating cited/citing references (e.g., browsing reference lists, using a citation index, setting up email alerts for references citing included studies).	P5 Line 120-122
Contacts	6	Indicate whether additional studies or data were sought by contacting authors, experts, manufacturers, or others.	P6 line 156-158
Other methods	7	Describe any additional information sources or search methods used.	None
SEARCH STRATEGIES			
Full search strategies	8	Include the search strategies for each database and information source, copied and pasted exactly as run.	See Appendix 1
Limits and restrictions	9	Specify that no limits were used, or describe any limits or restrictions applied to a search (e.g., date or time period, language, study design) and provide justification for their use.	P5 line 122-123
(Search filters	10	Indicate whether published search filters were used (as originally designed or modified), and if so, cite the filter(s) used.	P5 line 115
Prior work	11	Indicate when search strategies from other literature reviews were adapted or reused for a substantive part or all of the search, citing the previous review(s).	P5 line 117-120

Updates	12	Report the methods used to update the search(es) (e.g., rerunning searches, email alerts).	P4 line 111-P5 line112
Dates of searches	13	For each search strategy, provide the date when the last search occurred.	P4 line 111-P5 line112
**PEER REVIEW**			
Peer review	14	Describe any search peer review process.	P5 line 117-119
**MANAGING RECORDS**			
Total Records	15	Document the total number of records identified from each database and other information sources.	P8 line 223-230 & Figure 1
Deduplication	16	Describe the processes and any software used to deduplicate records from multiple database searches and other information sources.	P6 line 145-146

PRISMA-S: An Extension to the PRISMA Statement for Reporting Literature Searches in Systematic Reviews
Rethlefsen ML, Kirtley S, Waffenschmidt S, Ayala AP, Moher D, Page MJ, Koffel JB, PRISMA-S Group.
Last updated February 27, 2020.
